# Effectiveness of Universal Community Engagement Childhood Obesity Interventions at Improving Weight-Related and Behavioral Outcomes among Children and Adolescents: A Systematic Review and Meta-Analysis

**DOI:** 10.3390/nu16203465

**Published:** 2024-10-12

**Authors:** Pei Yong Koh, Joelle Yan Xin Chua, Pao Yi Chan, Shefaly Shorey

**Affiliations:** Alice Lee Centre for Nursing Studies, Yong Loo Lin School of Medicine, National University of Singapore, Singapore 117599, Singapore; koh.peiyong@u.nus.edu (P.Y.K.); e0006986@u.nus.edu (J.Y.X.C.); chan.pao.yi@u.nus.edu (P.Y.C.)

**Keywords:** community engagement, childhood obesity, children, adolescents, systematic review, meta-analysis

## Abstract

Background: Universal community engagement interventions can address childhood obesity. Objectives: This review aimed to evaluate the effectiveness of these interventions in improving body mass index (BMI) (primary outcome) as well as dietary choices and activity levels (secondary outcomes) among children and adolescents. Methods: Eight electronic databases were searched from inception dates to January 2024. A meta-analysis was conducted using the random-effect model, when appropriate; otherwise, the findings were narratively synthesized. Heterogeneity was determined by the I^2^ statistics and Cochran’s Q chi-squared test. The Cochrane ROB tool and the GRADE approach were used to assess the quality appraisal at the study and outcome levels, respectively. Results: Twenty-two studies were included in this review. The results showed that these interventions had a limited effect in improving children’s standardized BMI (BMI-z) scores post-intervention. A meta-analysis on BMI-z scores showed that the intervention group had a statistically non-significantly lower BMI-z score than the control group (MD = −0.02, 95%CI = [−0.07, 0.03], Z = 0.83, *p* = 0.40) at immediate post-intervention. It was also reported that universal community engagement interventions had a limited effect in improving children’s dietary choices and activity levels. Only the meta-analysis on children’s daily sugar-sweetened beverage intake measured using continuous data reported a statistically significant small effect favoring the intervention group (SMD = −0.25, 95%CI = [−0.38, −0.13], Z = 3.98, *p* < 0.0001) at immediate post-intervention. Conclusions: Universal community engagement interventions have the potential to address childhood obesity. Children and adolescents could benefit more from interventions that focus on implementing both environmental and behavioral changes, and interventions that include parental involvement.

## 1. Introduction

Childhood obesity is an ever-growing global epidemic [1,2]. According to the World Health Organization (WHO) [3,4], children and adolescents aged 2–19 years old are exhibiting more obesity-related behaviors, such as the increased intake of energy and reduced physical activity, which increases their risk of childhood obesity [5,6]. Studies have reported that around 7% of preschool children are obese, and an estimated 17.5–19.3% of children and adolescents aged 5–19 years old are obese [7,8]. Childhood obesity has been associated with detrimental effects on long-term physical, socioemotional, and psychological well-being [2,9,10,11,12]. Hence, childhood obesity is a global public health issue that warrants attention.

As childhood obesity is affected by biological, environmental, and social influences, community engagement interventions have the potential to address obesity among children and adolescents [13]. Community engagement interventions can be understood as interventions that have engaged community members (e.g., teachers, social leaders, and policymakers) to identify community assets and local determinants of childhood obesity to conduct culturally appropriate and feasible childhood obesity interventions [14]. Community engagement interventions often take place in community settings such as homes, schools, and community centers [15,16]. As these interventions often involve making adjustments to the children’s environment, such as making healthier food choices available in schools, they can be feasibly conducted on universal populations of children regardless of their obesity risk to maximize their benefits [17,18]. Therefore, researchers have been exploring the use of universal community engagement interventions for children aged >2 years old to tackle childhood obesity [19].

The body mass index standardized score (BMI-z), which refers to BMI adjusted for each child’s respective age and gender, has been widely used to assess children’s weight status and evaluate childhood obesity interventions [20]. As children’s dietary choices and activity levels significantly influence their weight status, they could also be used to assess the effectiveness of childhood obesity interventions. Studies have reported that consuming more fruits and vegetables and fewer sugar-sweetened beverages were associated with lower risks of childhood obesity [21,22]. Moreover, increased moderate-to-vigorous physical activity (MVPA) and lesser screen time also lowered children’s risk of obesity [23]. As one’s childhood dietary and activity habits often follow one into adulthood, childhood obesity interventions must encourage children to adopt positive lifestyle habits and reduce their lifetime obesity risk [24]. Since BMI-z scores, dietary choices, and activity levels are inter-related outcomes of childhood obesity, they should be examined simultaneously to evaluate childhood obesity interventions holistically.

While previous reviews have examined the effect of community engagement in childhood obesity interventions [25,26,27,28], only two of them have focused on universal interventions [25,28]. Moreover, previous reviews were conducted on children and adolescents aged 4–14 years old only [25], school-going children from the United States only [26], or both children and adults [27,28]. Additionally, while all existing reviews examined weight-related outcomes, only two reviews assessed behavioral outcomes such as self-reported physical activity and dietary habits (unhealthy and healthy food intake) [26,27]. As none of the previous reviews examined the effect of universal community engagement childhood obesity interventions in improving weight-related and behavioral outcomes among children and adolescents aged 2–19 years old, this study will seek to address this research gap.

### Aims

This review aimed to consolidate all available evidence regarding the effectiveness of universal community engagement childhood obesity interventions in improving BMI (primary outcome), as well as dietary choices and activity levels (secondary outcomes) among children and adolescents aged 2–19 years old. Dietary choices include their intake of fruits, vegetables, and sugar-sweetened beverages, while activity levels include MVPA and screen time.

## 2. Materials and Methods

This review was written in accordance with the Preferred Reporting Items for Systematic Reviews and Meta-Analyses (PRISMA) guidelines [29], and a protocol was submitted to PROSPERO (CRD42023455322).

### 2.1. Eligibility Criteria

The criteria in all of the following seven sections had to be met for studies to be included in this review: population, intervention, comparator, outcomes, study design, language, and publication status.

#### 2.1.1. Population

Children and adolescents aged 2–19 years old, regardless of obesity risk status, were included to examine children across their critical developmental stages (early childhood, middle childhood, and adolescence) [30]. Including both children at risk and children with no obesity risk facilitated the identification of protective factors towards obesity and the understanding of children’s normal developmental variability. Studies that specifically recruited only children who were overweight/obese or chronically ill and children with cognitive or developmental disabilities were excluded.

#### 2.1.2. Intervention

Universal community engagement interventions to improve childhood obesity were included. Community engagement interventions refer to interventions that involve community members’ input during at least one research stage (planning, design, implementation, data collection, and result analysis/dissemination) [14,31]. Such interventions offer a holistic approach to tackling childhood obesity by involving the relevant personnel in the community in creating healthier environments and promoting sustainable healthier behavioral changes among children [32]. These interventions could be conducted in any setting and delivered by any personnel using any mode of delivery.

#### 2.1.3. Comparator

Only studies with control groups that experienced no intervention, usual care (usual school curriculum/community activities), wait-list control, or placebo interventions (programs unrelated to childhood obesity) were included. Control groups that underwent any childhood obesity program not part of standard care/practice were excluded.

#### 2.1.4. Outcomes

The primary outcome was children’s BMI-z scores, and included studies had to assess these scores pre- and post-intervention. The secondary outcomes were dietary choices (fruit, vegetable, and sugar-sweetened beverage intake), activity levels (MVPA and screen time), and subsequent follow-up assessments of BMI-z scores. Not all of the included studies were required to assess any of these secondary outcomes. All of the outcomes could either be self-reported by the children and/or their parents/guardians or assessed by researchers using any data collection instrument, such as BMI calculators, food logs, activity logs, and questionnaires. Studies that reported children’s BMI in other formats (BMI only or BMI percentile) were excluded, as these values were not appropriately adjusted for their age and gender for statistical analyses [33]. Studies that did not report any post-test data were also excluded.

#### 2.1.5. Study Design

Only randomized controlled trials (RCTs), cluster RCTs, and clinical controlled trials (CCTs) were included. RCTs are the gold standard study design to examine causality [34], cluster RCTs can prevent intervention effects from spilling over between existing groups [35], and CCTs can serve as a good substitute when randomization is not feasible [36].

#### 2.1.6. Language and Publication Status

Only English-language peer-reviewed journal articles and gray literature (unpublished dissertations and conference proceedings with full texts) were included. Studies available only in abstract format and those lacking available full text were excluded, as they might lack the details needed for data synthesis in this review.

### 2.2. Study Selection

The search was conducted in eight electronic databases from their respective inception dates to January 2024 (PubMed, The Cochrane Library, Embase, CINAHL, PsycINFO, Scopus, Web of Science, and ProQuest Dissertations and Theses Global), using comprehensive search strategies developed under the guidance of an experienced medical librarian (Table S1, Supplementary File S1). Backward searching of reference lists from similar reviews and included studies was conducted to identify additional relevant studies. Authors of potentially relevant but inaccessible studies were contacted to obtain full texts. EndNote Version 20 was utilized for sorting all of the records and removing duplicates. All of the titles and abstracts were first screened against the eligibility criteria. Subsequently, selected articles underwent full-text screening to determine their eligibility. Two independent reviewers (P.Y. and C.T.) conducted the screening process, and discrepancies were settled via discussions.

### 2.3. Data Extraction

A customized data extraction form was used to extract important details from the included studies, such as the study design, the characteristics of the participants and interventions, community engagement research stages, the attrition rate, the use of intention-to-treat analysis, and the outcomes measured. The form was pilot-tested on five random included studies by two independent reviewers (P.Y and C.T.) to ensure its adequacy, and no further amendments were made [37]. The event and total values of the review’s outcomes were extracted from studies that reported outcomes using dichotomous data. The mean and standard deviation (SD) values of the review’s outcomes were also extracted from studies that reported outcomes using continuous data. Appropriate formulae and online calculators were used to transform the data into the appropriate formats for analyses [37,38,39]. Two independent reviewers (P.Y and C.T.) conducted the extraction process, and disagreements were discussed among all authors.

### 2.4. Quality Appraisal

The Cochrane Risk of Bias tool Version 1 was used to assess five types of biases in the following seven domains: random sequence generation (selection bias), allocation concealment (selection bias), blinding of participants and personnel (performance bias), blinding of outcome assessment (detection bias), incomplete outcome data (attrition bias), selective reporting (reporting bias), and other sources of bias [40]. As the BMI-z scores and this review’s secondary outcomes (dietary choices and activity levels) were assessed via bio-physiological measurements and self-reported questionnaires, respectively, they were assessed separately for the blinding of the outcome assessment domain. The overall bias rating for each study was determined by the worst score it received for any domain [41]. All of the studies, regardless of their quality appraisal scores, were included in this review to improve this review’s rigor [42,43]. Publication bias was examined using funnel plots for any outcome that had at least 10 trials in the forest plot [44].

Quality appraisal at the outcome level was conducted using the Grades of Recommendation, Assessment, Development, and Evaluation (GRADE) approach. Ratings were affected by the following five factors: individual studies’ risk of bias, inconsistency, directness of evidence, precision of effect estimates, and publication bias [37]. Each outcome was rated individually using GRADEpro software (https://www.gradepro.org/, accessed on 30 January 2024) [45]. The quality appraisal process at the study and outcome levels was conducted by two independent reviewers (P.Y. and C.T.), and discrepancies in judgement were discussed with the other authors.

### 2.5. Data Synthesis

The included studies’ and interventions’ characteristics were narratively summarized. Under the random effect model, Review Manager software version 6.6 was used to conduct meta-analyses to pool data regarding the same outcomes [37]. Using the inverse-variance method, the mean differences (MDs) with 95% confidence intervals (CIs) were calculated for the outcome of the BMI-z scores [37]. As the studies used different scales to report children’s dietary choices and activity levels, standardized mean differences (SMDs) and 95% CIs were calculated for the review’s secondary outcomes under the inverse-variance method [37]. The SMD effect sizes were interpreted as very small (0.1), small (0.2), medium (0.5), large (0.8), very large (1.2), and huge (2.0) [46]. Studies that measured continuous outcomes using change-from-baseline scores and scores at post-intervention time points were pooled for meta-analyses [47]. Moreover, for studies that reported results using dichotomous data, the risk ratio (RR) and 95% CIs were used as the effect measure under the Mantel–Haenszel method [37]. As Crespo et al. (2012) had three intervention arms [19], the number of children in the control group was randomly divided to facilitate pair-wise comparison with each intervention arm and to prevent unit-of-analysis errors [48].

Heterogeneity was assessed using the I² statistic and Cochran Q chi-squared test. The I^2^ values were categorized as low importance, moderate, substantial, and considerable according to their values of ≤40%, 30–60%, 50–90%, and 75–100%, respectively [37]. Statistically significant heterogeneity of the chi-squared test was recognized when its corresponding *p*-value < 0.10 [37]. When the heterogeneity levels were considerable, sensitivity analyses were conducted to identify outliers—studies that significantly differed from the others based on participants’ characteristics, intervention type, or psychometric scales [37]. Subgroup analyses were also conducted to investigate the sources of heterogeneity and examine the effect of specific variables on the review outcomes [37]. The variables examined were the number of community engagement research stages and the type of intervention.

Narrative synthesis for the BMI-z scores was conducted for studies that reported significant baseline differences in BMI-z scores, waist circumference, or obesity prevalence between the intervention and control groups [49,50,51,52,53], and BMI-z data which could not be transformed into mean and SD values [16]. As only one study reported MVPA using dichotomous data [49], only one study reported small screen time using continuous data [54], and only one study reported the intake of fruits, vegetables, and sugar-sweetened beverages over three days [52], the results of these outcomes were narratively summarized. Moreover, two studies had missing post-intervention data, such as a missing sample size, and had all of their outcomes narratively synthesized [55,56]. Follow-up assessments of the review outcomes were only conducted by three studies at varying durations, and hence they were narratively synthesized as well [19,49,57].

## 3. Results

### 3.1. Search Outcomes

**Figure 1:**
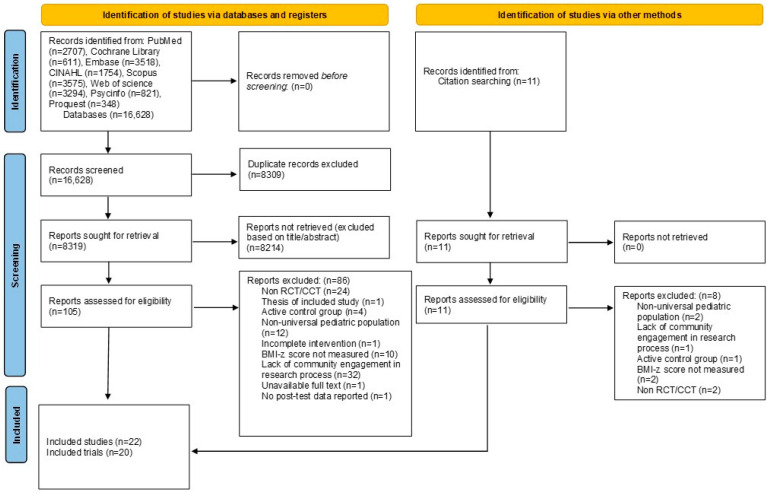
PRISMA flow diagram.

In total, 16,628 records were retrieved from the eight listed electronic databases and citation searching. After the removal of duplicates and irrelevant titles/abstracts, 116 records had their full texts examined. The full-text screening process excluded another 94 articles, resulting in 22 included studies [15,16,19,49,50,51,52,53,54,55,56,57,58,59,60,61,62,63,64,65,66,67]. With Folta et al. (2013) [54] and Kattelmann et al. (2019) [61] reporting the secondary outcomes of the main studies reported in Economos et al. (2013) [16] and White et al. (2019) [66], respectively, the 22 studies reported 20 trials. The search outcomes are presented using the PRISMA flow diagram (Figure 1).

### 3.2. Characteristics of Included Studies

The 20 trials were conducted in the following countries: Australia (n = 3), the United States (n = 10), New Zealand (n = 1), Denmark (n = 1), Fiji (n = 1), Spain (n = 1), Sweden (n = 1), Tonga (n = 1), and the United Kingdom (n = 1). Five trials were RCTs, five were cluster RCTs, and the remaining ten were CCTs. The children and adolescents ranged from preschool age to high school age. Six trials aimed to influence children and adolescents to make behavioral changes (e.g., increased physical activity) [15,49,56,57,66,67], while five trials aimed to change children’s physical and social environments [19,50,51,59,62], and the remaining nine conducted a combination of behavioral and environmental interventions. Community members were engaged in the following number of research stages: one stage (n = 1), two stages (n = 5), three stages (n = 12), and four stages (n = 2). Lastly, all trials, except Wong et al. (2016) [67], utilized a theoretical/conceptual framework for intervention development. More details regarding the included studies are shown in Table S2 (Supplementary File S1).

### 3.3. Quality Appraisal at Study Level

The Cochrane Risk of Bias tool rated 15 studies as having a high overall risk, 6 studies as having an unclear overall risk, and 1 study as having a low overall risk. The two independent reviewers had an inter-rater agreement of approximately 95% and a Cohen’s kappa value of 0.91. The ratings are shown in Table 1.

### 3.4. BMI-z Scores

A meta-analysis was conducted for the 12 studies that assessed BMI-z scores at immediate post-intervention [15,19,57,58,59,60,62,63,64,65,66,67]. The results showed that the intervention group had a statistically non-significantly lower BMI-z score than the control group (MD = −0.02, 95%CI = [−0.07, 0.03], Z = 0.83, *p* = 0.40), with a low statistical heterogeneity (I^2^ = 20%, *p* = 0.23) (Figure S1, Supplementary File S2).

The subgroup analyses according to the number of community engagement research stages and the type of intervention showed statistically non-significant subgroup differences (*p* ≥ 0.05) (Figure S2, Supplementary File S2).

### 3.5. Dietary Choices (Fruit, Vegetable, Sugar-Sweetened Beverage Intake)

#### 3.5.1. Fruit Intake (Dichotomous Data)

A meta-analysis of the three studies [50,51,62] that assessed fruit intake using dichotomous data at immediate post-intervention showed that, although not statistically significant, the intervention group had a 0.06 less chance of meeting their daily fruit recommendations as compared to the control group (RR = 0.94, 95%CI = [0.87, 1.02], Z = 1.57, *p* = 0.12), with low statistical heterogeneity (I^2^ = 31%, *p* = 0.24) (Figure S3, Supplementary File S2). No subgroup analyses were conducted, as all of the studies conducted interventions focused on environmental change only and had community engagement in three research stages.

#### 3.5.2. Fruit Intake (Continuous Data)

A meta-analysis of three studies [57,60,65] measuring daily fruit intake at immediate post-intervention reported a statistically non-significant negligible effect favoring the control group (SMD = −0.03, 95%CI = [−0.24, 0.17], Z = 0.31, *p* = 0.75), with low statistical heterogeneity (I^2^ = 22%, *p* = 0.28) (Figure S4, Supplementary File S2).

The subgroup analyses according to the number of community engagement research stages and type of intervention showed statistically non-significant subgroup differences (*p* ≥ 0.05) (Figure S5, Supplementary File S2).

#### 3.5.3. Vegetable Intake (Dichotomous Data)

A meta-analysis of the three studies [50,51,62] that assessed vegetable intake using dichotomous data at immediate post-intervention showed that the intervention group had a statistically significantly 0.08 less chance of meeting their daily vegetable recommendations as compared to the control group (RR = 0.92, 95%CI = [0.89, 0.96], Z = 4.03, *p* < 0.0001), with low statistical heterogeneity (I^2^ = 0%, *p* = 0.38) (Figure S6, Supplementary File S2). No subgroup analyses were conducted, as all of the studies conducted interventions focused on environmental change only and had community engagement in three research stages.

#### 3.5.4. Vegetable Intake (Continuous Data)

A meta-analysis of three studies [57,60,65] measuring daily vegetable intake at immediate post-intervention reported a statistically non-significant negligible effect favoring the control group (SMD = −0.02, 95%CI = [−0.23, 0.19], Z = 0.18, *p* = 0.86), with low statistical heterogeneity (I^2^ = 27%, *p* = 0.26) (Figure S7, Supplementary File S2).

The subgroup analyses according to the number of community engagement research stages and type of intervention showed statistically non-significant subgroup differences (*p* ≥ 0.05) (Figure S8, Supplementary File S2).

#### 3.5.5. Sugar-Sweetened Beverage Intake (Dichotomous Data)

A meta-analysis of the four studies [50,51,59,62] that assessed sugar-sweetened beverage intake using dichotomous data at immediate post-intervention showed that, although not statistically significant, the intervention group had a 0.07 greater chance of staying within the limits of their daily sugar-sweetened beverage recommendations as compared to the control group (RR = 0.93, 95%CI = [0.82, 1.05], Z = 1.21, *p* = 0.22), with statistically significant substantial heterogeneity (I^2^ = 88%, *p* < 0.0001) (Figure S9, Supplementary File S2). Sensitivity analyses were conducted but no outlier was identified. No subgroup analyses were conducted, as all of the studies conducted interventions focused on environmental change only and had community engagement in three research stages.

#### 3.5.6. Sugar-Sweetened Beverage Intake (Continuous Data)

A meta-analysis of three studies [19,54,57] measuring daily sugar-sweetened beverage intake at immediate post-intervention reported a statistically significant small effect favoring the intervention group (SMD = −0.25, 95%CI = [−0.38, −0.13], Z = 3.98, *p* < 0.0001), with low statistical heterogeneity (I^2^ = 0%, *p* = 0.84) (Figure S10, Supplementary File S2).

The subgroup analyses according to the number of community engagement research stages and type of intervention showed statistically non-significant subgroup differences (*p* ≥ 0.05) (Figure S11, Supplementary File S2).

### 3.6. Activity Levels (MVPA and Screen Time)

#### 3.6.1. MVPA (Continuous Data)

A meta-analysis of four studies [49,60,61,67] measuring daily MVPA at immediate post-intervention reported no intervention effect (SMD = −0.00, 95%CI = [−0.10, 0.10], Z = 0.05, *p* = 0.96), with low statistical heterogeneity (I^2^ = 0%, *p* = 0.56) (Figure S12, Supplementary File S2).

The subgroup analyses according to the number of community engagement research stages and type of intervention showed statistically non-significant subgroup differences (*p* ≥ 0.05) (Figure S13, Supplementary File S2).

#### 3.6.2. Small Screen Time (Dichotomous Data)

In this review, small screen time referred to time spent viewing the television. A meta-analysis of four studies [50,51,59,62] that assessed small screen time using dichotomous data at immediate post-intervention showed that, although not statistically significant, the intervention group had a 0.05 less chance of meeting their daily small screen time recommendations as compared to the control group (RR = 0.95, 95%CI = [0.86, 1.05], Z = 1.01, *p* = 0.31), with statistically significant substantial heterogeneity (I^2^ = 86%, *p* = 0.0001) (Figure S14, Supplementary File S2). Sensitivity analyses were conducted but no outlier was identified. No subgroup analyses were conducted, as all of the studies conducted interventions focused on environmental change only and had community engagement in three research stages.

#### 3.6.3. Electronic Game Time (Dichotomous Data)

In this review, the electronic game time refers to time spent playing video/computer games. A meta-analysis of four studies [50,51,59,62] that assessed electronic game time using dichotomous data at immediate post-intervention showed that, although not statistically significant, the intervention group had a 0.07 less chance of meeting their daily electronic game time recommendations as compared to the control group (RR = 0.93, 95%CI = [0.81, 1.07], Z = 0.98, *p* = 0.33), with statistically significant substantial heterogeneity (I^2^ = 90%, *p* < 0.00001) (Figure S15, Supplementary File S2). Sensitivity analyses were conducted but no outlier was identified. No subgroup analyses were conducted, as all of the studies conducted interventions focused on environmental change only and had community engagement in three research stages.

### 3.7. Quality Appraisal at Outcome Level

A funnel plot of the standard errors (SEs) against the MDs was produced for the BMI-z scores that had 12 studies in its forest plot. The distribution of included study results was relatively even, with no systematic tendency for studies reporting negative or positive results to be over-represented or under-represented. Thus, a visual inspection of the plot revealed no observed pattern of publication bias (Figure S16, Supplementary File S2).

The GRADE approach rated the outcome of MVPA (continuous data) to be of low quality, and the remaining review outcomes were rated as very low quality. This is primarily because most of the included studies have a high overall risk due to the lack of randomization methods (selection bias), and the secondary outcomes are mostly self-reported (desirability bias). Detailed explanations for these ratings can be found in Table S3 (Supplementary File S1).

### 3.8. Narrative Synthesis

#### 3.8.1. BMI-z Scores

Buch-Andersen et al. (2021) reported that the control group had a statistically significant increase in BMI-z scores from baseline as compared to the intervention group [53]. Adab et al. (2018) and Kremer et al. (2011) reported similar BMI-z scores between both groups at post-intervention, although their control groups had higher BMI-z scores, and more children were categorized as overweight and underweight at baseline, respectively [49,50]. On the other hand, Malakellis et al. (2017) reported statistically significantly lower BMI-z scores in the intervention group at both pre-and post-intervention time points than the control group [51]. Taylor et al. (2007) reported statistically significantly lower BMI-z scores in the intervention group than in the control group at post-intervention after adjusting for baseline differences [52]. Similar BMI-z scores were noted between the intervention and control groups of Elinder et al. (2012) and Tomayko et al. (2019) at post-intervention [55,56]. At post-intervention, the intervention group of Economos et al. (2013) had a statistically significant decrease in BMI-z scores compared to baseline after adjusting for covariates, while the control group did not [16]. No follow-up assessments were conducted by these eight studies [16,49,50,51,52,53,55,56].

Follow-up assessments of BMI-z scores in three studies were measured at various durations [19,49,57]. No statistically significant differences in BMI-z scores between the intervention and control groups were reported at the 18-month follow-up in Adab et al. (2018) [49], and the 1- and 2-year follow-up in Crespo et al. (2012) [19]. Conversely, Black et al. (2010) [57] reported a statistically non-significant decrease in BMI-z scores at the 14-month follow-up for the intervention group compared to its post-intervention scores [57].

#### 3.8.2. Dietary Choices

At post-intervention, Tomayko et al. (2017) reported that the intervention group consumed statistically non-significantly fewer sugar-sweetened beverages daily than the control group [56], while Elinder et al. (2012) reported similar proportions of children who stayed within their daily recommended sugar-sweetened beverage limitation across both groups [55]. Conversely, Taylor et al. (2007) reported that the intervention group consumed statistically significantly more fruits and fewer sugar-sweetened beverages, but a similar intake of vegetables, over three days than the control group at post-intervention [52]. No follow-up assessments were conducted by these three studies [52,55,56].

At 14 months follow-up, Black et al. (2010) reported statistically non-significant decreases in the daily fruit, vegetable, and sugar-sweetened beverage intake for the intervention group compared to the post-intervention intake [57]. Crespo et al. (2012) reported similar daily sugar-sweetened beverage intake between the intervention and control groups at the 1- and 2-year follow-up [19].

#### 3.8.3. Activity Levels

At post-intervention, a similar proportion of children from the intervention and control groups reported meeting the daily small screen time recommendations in Elinder et al. (2012), while the duration spent on daily small screen time was similar between the intervention and control groups in Folta et al. (2013). These two studies did not conduct any follow-up assessments [54,55].

At the 18-month follow-up, Adab et al. (2018) reported similar levels of MVPA (duration and proportion of children who achieve ≥60 min daily) in both intervention and control groups [49].

## 4. Discussion

This review examined the effect of universal community engagement childhood obesity interventions in improving BMI-z scores, dietary choices, and activity levels among children and adolescents. Results from the meta-analyses and narrative synthesis showed that these interventions had a limited effect in improving children’s BMI-z scores at post-intervention. A similar reduction in BMI-z scores was reported by a previous review; however, the previous review reported statistically significant findings instead [28]. The difference in the results could be due to the previous review’s limited database search, which only searched across four databases [28]. This narrow scope may have led to the omission of studies that met the eligibility criteria. The limited improvement in children’s BMI-z scores could also be attributed to the recruitment of universal pediatric populations, where most children and adolescents had a healthy weight status at baseline [68]; as a result, there was less room for improvement in their BMI-z scores during the intervention, leading to a floor effect [69]. To obtain a more accurate understanding of the effectiveness of these universal community engagement interventions on childhood obesity, future researchers could assess the overweight/obesity prevalence among the study participants in addition to their BMI-z scores [68].

The current results also reported that universal community engagement interventions had a limited effect in improving children’s dietary choices and activity levels. Only the meta-analysis on children’s daily sugar-sweetened beverage intake measured using continuous data showed a significant improvement [19,54,57]. The three studies that examined daily sugar-sweetened beverage intake using continuous data conducted interventions included home-based components, unlike the other studies, which assessed children’s other dietary choices and activity levels [19,54,57]. The similar effectiveness of home-based components in childhood obesity interventions was echoed by previous research [70]. By educating parents and children on how to improve their lifestyle habits, such as selecting healthier alternatives to sugar-sweetened beverages, and increasing their engagement in physical activity, home-based components can provide personalized instructions for families to address childhood obesity [13,71]. As parents can significantly influence their children’s dietary habits and lifestyle behaviors, future childhood obesity interventions could aim to engage parents as change agents to improve children’s behaviors [71,72]. Despite the limited effects on children’s BMI-z scores, dietary choices, and activity levels, this review showed that universal community engagement interventions were feasible in addressing childhood obesity. Considering that childhood obesity is affected by biological, environmental, and sociocultural factors [13], and these interventions can address children’s environmental and sociocultural influences, future research could be encouraged to further explore these interventions. The high heterogeneity reported for some dietary and lifestyle behaviors, such as sugar-sweetened beverage intake and screen time, could be due to the differing definitions of acceptable sugar-sweetened beverage intake and screen time across the included studies. Hence, future research could adopt standardized guidelines, such as those provided by the WHO [73,74,75], to ensure comparability in the measurement and evaluation of intervention outcomes.

The current findings showed that the number of research stages that community members were involved in did not affect the effectiveness of the community engagement interventions. This could be because all of the included studies involved community members in at least one of the intervention development phases, such as the planning and design phases or implementation phases, of the intervention. Being involved in the preparation/implementation phases would allow community members to determine key features of the intervention (content, layout, and format), which would further help researchers to develop programs tailored to address the local communities’ needs and ensure that the interventions were executed as intended [76]. Only two included studies involved community members during the data collection phase [55,61], and none of the included studies involved community members in the data analysis phase. Community members could also contribute meaningfully during the data collection and analysis phases by helping to collect more objective assessments of children’s behaviors through the use of observation checklists as opposed to self-reported questionnaires, and refining the interventions after the data analysis to improve their effectiveness [76]. Hence, researchers could seek to engage community members in all five research phases (planning, design, implementation, data collection, and data analysis/dissemination) for future childhood obesity interventions so to benefit from the members’ expertise in local communities.

This review found that interventions involving environmental changes, behavioral changes, or a combination of both were all feasible methods of addressing childhood obesity. Interventions that cause environmental changes focused on manipulating the children’s environments (e.g., home, school, and community facilities) to influence them to engage in healthy behaviors [77]. Such changes include the removal of soft drinks from school vending machines and adding more exercise facilities in the community areas (e.g., parks and community centers) [62]. These changes would make it more convenient and accessible for children and adolescents to engage in healthy behaviors and decrease their risk of obesity [77]. In contrast, interventions that cause behavioral changes focused on motivating children and adolescents to engage in healthy behaviors (e.g., consuming more fruits and vegetables and engaging in more physical activity) [78]. They typically involved educational programs that teach children and adolescents how to plan well-balanced meals and engage in more physical activity [66,67]. Since obesity is influenced by one’s environmental and social influences [13], future community engagement interventions could aim to improve both the children’s environment and behaviors concurrently to maximize their impact on the children’s lifestyles and weight status [77].

Similar to a previous review [28], the current results showed that universal community engagement interventions had limited effectiveness in improving children’s BMI-z scores, dietary choices, and activity levels at 1–2 years follow-up. Due to social pressures to consume junk food and the lack of parental supervision, children and adolescents could have easily lost their motivation to continue engaging in healthy lifestyle choices after the intervention [79]. Although community assets such as new exercise facilities and vending machines containing healthier food choices could have been set up during the intervention for the children’s long-term use, children might have lost the motivation to utilize them after the intervention [80]. As engaging in healthy lifestyles involves continuous daily efforts, researchers would need to collaborate with local community leaders to establish plans for these leaders to take over the intervention programs in a sustainable manner after the research study has been completed [81,82]. Hence, future researchers could aim to foster community leadership and promote greater community ownership to help local communities continue the interventions during the post-research period and to achieve long-term community health goals [81,82]. However, as only 3 out of the 20 included trials conducted follow-up assessments in this review [19,49,57], future studies would need to conduct follow-up assessments to evaluate the sustainability of the interventions’ effect on childhood obesity.

### 4.1. Limitations

Only English-reported studies were included, potentially causing publication bias. With most of the studies conducted in predominantly Caucasian countries, the current findings could not be generalized to adolescents. As few included studies were conducted on children below five years old, the current results could not accurately represent this group of young children. Since children’s dietary choices and activity levels were often self-reported by the children themselves or their parents, there was also a risk of social desirability bias. Some meta-analyses and subgroup analyses were inadequately powered (e.g., fruit intake and vegetable intake), which could limit the reliability and validity of the results and potentially result in false-negative findings [83]. With only 3 out of 20 trials conducting follow-up assessments, the sustainability of universal community engagement interventions could not be determined. Moreover, this review’s analysis of children’s dietary intake was limited to fruits, vegetables, and sugar-sweetened beverages, and did not explore their intake of other important food groups such as legumes, whole grains, nuts, and seeds [84]. This was due to a lack of readily available data regarding children’s intake of legumes, whole grains, nuts, and seeds across the included studies, which limited this review’s analyses to only fruits, vegetables, and sugar-sweetened beverages. Hence, this affected the current review’s ability to holistically evaluate the effect of community engagement interventions on children’s diets.

Given that most of the included studies (n = 17) were conducted in high-income countries, the current findings might not reflect the effect of community engagement interventions on childhood obesity globally, especially those from lower-income countries, where contextual factors, cultural differences, and resource constraints could significantly impact the effectiveness and implementation of such interventions. This suggests the need for further research to include a broader range of settings to ensure that the interventions are adaptable and relevant across different socioeconomic contexts. Lastly, as the GRADE approach rated the review outcomes as either low or very low quality, there is limited to very little confidence in the effect estimate of this review, and the true effect is likely to be very different from this review’s results.

### 4.2. Implications for Future Research and Practice

Overall, although the current findings showed that universal community engagement interventions had a limited effect (statistically non-significant results or small effect sizes only) in improving children’s BMI-z scores, dietary choices, and activity levels, this review has shown that these interventions are feasible and viable ways to address childhood obesity. With the ability to reach out to the general population of children and modify their environment to encourage long-term healthy behavioral changes, universal community engagement interventions have the potential to influence the upcoming global and societal issues related to childhood obesity. Thus, researchers could further explore these interventions and seek to collaborate with local community leaders to implement these interventions as part of the public healthcare system to benefit children and adolescents. The assessment of both BMI-z scores and the overweight/obesity prevalence among study participants could allow the interventions’ effect on children’s weight status to be measured more accurately. To address issues of high heterogeneity, future studies could adhere to the standard recommended guidelines set by the WHO regarding dietary and lifestyle behaviors, such as the appropriate sugar-sweetened beverage intake and screen time. As parents play a crucial role in children’s dietary habits and lifestyle behaviors, future studies on childhood obesity could aim to incorporate more parental involvement. Considering that children and adolescents will benefit from cultivating positive behaviors to lead healthy lifestyles and having a conducive environment to engage in healthy lifestyle behaviors, future interventions could aim to improve both the children’s environment and behaviors simultaneously. Future research could also explore the effect of these interventions on children’s diets more holistically by measuring their intake of important food groups beyond fruits and vegetables, such as legumes, nuts, seeds, and grains. To better leverage the expertise of local community members, researchers could seek to engage them in all five research phases (planning, design, implementation, data collection, and data analysis/dissemination) of future studies. After the completion of research studies, local community leaders would need to continue implementing similar interventions (e.g., sports programs, cooking classes, and dietary lessons) for children and adolescents so to continue motivating them to engage in healthy lifestyle behaviors. As sociocultural factors affect childhood obesity [13], more studies could be conducted in Asia, Africa, and the Middle East to obtain a more global understanding of childhood obesity. With children as young as 2 years old being at risk for obesity, future studies could be conducted among younger children (2–5 years old) [6]. Lastly, future studies would need to conduct follow-up assessments to better understand the sustainability of the interventions’ effect.

## 5. Conclusions

In conclusion, this review demonstrated that universal community engagement interventions are viable methods for addressing childhood obesity. The current findings indicate that children and adolescents can benefit from interventions that include both environmental and behavioral changes, as well as those involving their parents’ engagement. Future research should involve closer collaboration with local community leaders throughout all phases of the research process—planning, design, implementation, data collection, and analysis. These leaders should also be equipped with the skills needed to continue implementing similar interventions after the research concludes. Moreover, further studies are needed to assess the long-term effects of these interventions. Despite its limitations, this review identified potentially beneficial components of universal community engagement interventions for childhood obesity and highlighted research gaps for future studies to address.

## Figures and Tables

**Table 1:** Risk of bias ratings for all included studies.

Study	Selection Bias: Random Sequence Generation	Selection Bias: Allocation Concealment	Performance Bias: Blinding of Participants and Personnel	Detection Bias: Blinding of Outcome Assessment (BMI-z Score)	Detection Bias: Blinding of Outcome Assessment (Secondary Outcomes)	Attrition Bias: Incomplete Outcome Data	Reporting Bias: Selective Reporting	Other Bias	Overall Bias
Adab et al., 2018 [49]	Low risk	Low risk	Low risk	Low risk	Low risk	Low risk	Low risk	Low risk	Low risk
Black et al., 2010 [57]	Unclear risk	Unclear risk	Unclear risk	Low risk	Low risk	Unclear risk	Low risk	Low risk	Unclear risk
Buch-Andersen et al., 2021 [53]	High risk	Unclear risk	Low risk	Unclear risk	Not applicable (no secondary outcome assessed)	Low risk	Low risk	Low risk	High risk
Crespo et al., 2012 [19]	Unclear risk	Unclear risk	Low risk	Low risk	Low risk	Low risk	Low risk	Low risk	Unclear risk
Davis et al., 2016 [58]	Low risk	Unclear risk	Low risk	Unclear risk	Not applicable (no secondary outcome assessed)	Unclear risk	Unclear risk	Low risk	Unclear risk
Economos et al., 2013 [16]	High risk	Unclear risk	Low risk	Unclear risk	Not applicable (no secondary outcome assessed)	Low risk	Low risk	Low risk	High risk
Elinder et al., 2012 [55]	High risk	Unclear risk	Low risk	Unclear risk	Low risk	Low risk	Low risk	Low risk	High risk
Folta et al., 2013 [54]	High risk	Unclear risk	Low risk	Not applicable (no primary outcome assessed)	Low risk	Low risk	Low risk	Low risk	High risk
Fotu et al., 2011 [59]	High risk	Unclear risk	Low risk	Unclear risk	Low risk	High risk	Low risk	Low risk	High risk
Fulkerson et al., 2022 [60]	Low risk	Unclear risk	High risk	High risk	Low risk	Low risk	Unclear risk	Low risk	High risk
Gómez et al., 2018 [15]	Low risk	Unclear risk	Low risk	Unclear risk	Low risk	Low risk	High risk	Low risk	High risk
Kattelmann et al., 2019 [61]	Low risk	Unclear risk	Unclear risk	Not applicable (no primary outcome assessed)	Low risk	Low risk	Low risk	Low risk	Unclear risk
Kremer et al., 2011 [50]	High risk	Unclear risk	Low risk	High risk	Low risk	High risk	Low risk	Unclear risk	High risk
Malakellis et al., 2017 [51]	High risk	High risk	Low risk	High risk	High risk	High risk	Low risk	Unclear risk	High risk
Millar et al., 2011 [62]	High risk	Unclear risk	Low risk	Unclear risk	Low risk	High risk	Low risk	Unclear risk	High risk
Peña et al.,2021 [63]	High risk	Unclear risk	Low risk	Low risk	Not applicable (no secondary outcome assessed)	Low risk	Low risk	Low risk	High risk
Sanigorski et al., 2008 [64]	High risk	Unclear risk	Low risk	Unclear risk	Low risk	Low risk	Low risk	Unclear risk	High risk
Scherr et al., 2017 [65]	Low risk	Unclear risk	Low risk	Low risk	Low risk	Low risk	Low risk	Low risk	Unclear risk
Taylor et al., 2007 [52]	High risk	Unclear risk	Low risk	Unclear risk	Unclear risk	High risk	Low risk	Low risk	High risk
Tomayko et al., 2019 [56]	Low risk	Unclear risk	High risk	Unclear risk	High risk	Unclear risk	High risk	High risk	High risk
White et al., 2019 [66]	Low risk	Unclear risk	Unclear risk	Unclear risk	Not applicable (no secondary outcome assessed)	Low risk	Low risk	Low risk	Unclear risk
Wong et al., 2016 [67]	High risk	Unclear risk	Low risk	Unclear risk	Low risk	High risk	Low risk	Unclear risk	High risk

## Data Availability

The authors confirm that the data supporting the findings of this study are available within the article and its Supplementary Materials.

## Supplementary Materials

The following supporting information can be downloaded at: https://www.mdpi.com/article/10.3390/nu16203465/s1, Table S1: Search strategies for all databases; Table S2: Table of included studies’ characteristics; Table S3: GRADE summary of the evidence table; Figure S1: Forest plot of BMI-z scores at post-intervention in the 12 included studies; Figure S2: Forest plots of subgroup analyses on BMI-z scores at post-intervention according to (a) the number of community engagement research stages and (b) the type of intervention; Figure S3: Forest plot of fruit intake (dichotomous data) at post-intervention in three of the included studies; Figure S4: Forest plot of fruit intake (continuous data) at post-intervention in three of the included studies; Figure S5: Forest plot of fruit intake (continuous data) at post-intervention according to (a) the number of community engagement research stages and (b) the type of intervention; Figure S6: Forest plot of vegetable intake (dichotomous data) at post-intervention in three of the included studies; Figure S7: Forest plot of vegetable intake (continuous data) at post-intervention in three of the included studies; Figure S8: Forest plot of vegetable intake (continuous data) at post-intervention according to (a) the number of community engagement research stages and (b) the type of intervention; Figure S9: Forest plot of sugar-sweetened beverage intake (dichotomous data) at post-intervention in four of the included studies; Figure S10: Forest plot of sugar-sweetened beverage intake (continuous data) at post-intervention in three of the included studies; Figure S11: Forest plot of sugar-sweetened beverage intake (continuous data) at post-intervention according to (a) the number of community engagement research stages and (b) the type of intervention; Figure S12: Forest plot of MVPA (continuous data) at post-intervention in four of the included studies; Figure S13: Forest plot of MVPA (continuous data) at post-intervention according to (a) the number of community engagement research stages and (b) the type of intervention; Figure S14: Forest plot of small screen time (dichotomous data) at post-intervention in four of the included studies; Figure S15: Forest plot of electronic game time (dichotomous data) at post-intervention in four of the included studies; Figure S16. Funnel plot of BMI-z scores using standard errors (SEs) against the mean difference (MD): 12 studies (lower MD values indicate better outcomes).
