# Supplementary material for: Effectiveness of Universal Community Engagement Childhood Obesity Interventions at Improving Weight-Related and Behavioral Outcomes among Children and Adolescents: A Systematic Review and Meta-Analysis
Source: Nutrients. 2024 Oct 12;16(20):3465. doi: 10.3390/nu16203465 (PMC11510657; doi:10.3390/nu16203465)
Supplement: Supplementary file 1 [file nutrients-16-03465-s001.zip › Supplementary File S1.pdf]

Supplementary file S1: Tables S1-S3

Table S1. Search strategies for all databases

Database	Search strategy
Embase	'child'/exp OR 'adolescent'/exp OR 'preschool child'/exp OR child*:ti,ab,kw OR adolesce*:ti,ab,kw OR juvenile*:ti,ab,kw OR pediatr*:ti,ab,kw OR paediatr*:ti,ab,kw OR toddler*:ti,ab,kw OR preschool*:ti,ab,kw OR preteen*:ti,ab,kw OR teen*:ti,ab,kw OR youth*:ti,ab,kw OR kid*:ti,ab,kw<br><br>AND<br><br>'childhood obesity'/exp OR 'obesity'/exp OR overweigh*:ti,ab,kw OR obes*:ti,ab,kw<br><br>AND<br><br>'community participation'/exp OR 'participatory research'/exp OR 'participatory action research'/exp OR “CBPR”:ti,ab,kw OR ((participat* NEAR/3 research*):ti,ab,kw) OR ((communit* NEAR/3 (participat* OR research* OR act* OR engag* OR partner* OR coalition* OR involv* OR contribut* OR cooperat* OR inform* OR consult* OR empower* OR effort* OR base* or basis*)):ab,ti,kw)<br><br>**<u>Legend</u>**<br>*: truncation of word<br>ab: abstract<br>exp: exploded<br>kw: keywords<br>NEAR/n: words must be within n words inclusive of each other in the record<br>ti: title
Pubmed	"Child"[MeSH Terms] OR "child, preschool"[MeSH Terms] OR "Adolescent"[MeSH Terms] OR "pediatr*"[Title/Abstract] OR "paediatr*"[Title/Abstract] OR "child*"[Title/Abstract] OR "toddler*"[Title/Abstract] OR "preschool*"[Title/Abstract] OR "preteen*"[Title/Abstract] OR "teen*"[Title/Abstract] OR

"adolesce*"[Title/Abstract] OR "youth*"[Title/Abstract] OR "juvenile*"[Title/Abstract] OR "kid"[Title/Abstract] OR "kids"[Title/Abstract]

AND

"Pediatric Obesity"[MeSH Terms] OR "Obesity"[MeSH Terms] OR "Overweight"[MeSH Terms] OR "overweigh*"[Title/Abstract] OR "obes*"[Title/Abstract]

AND

"Community Participation"[MeSH Terms] OR "Community-Based Participatory Research"[MeSH Terms] OR "CBPR"[Title/Abstract] OR "participate research"[Title/Abstract:~3] OR "participate researches"[Title/Abstract:~3] OR "participate researched"[Title/Abstract:~3] OR "participate researching"[Title/Abstract:~3] OR "participates research"[Title/Abstract:~3] OR "participates researches"[Title/Abstract:~3] OR "participates researched"[Title/Abstract:~3] OR "participates researching"[Title/Abstract:~3] OR "participating research"[Title/Abstract:~3] OR "participating researches"[Title/Abstract:~3] OR "participating researched"[Title/Abstract:~3] OR "participating researching"[Title/Abstract:~3] OR "participated research"[Title/Abstract:~3] OR "participated researches"[Title/Abstract:~3] OR "participated researched"[Title/Abstract:~3] OR "participated researching"[Title/Abstract:~3] OR "participatory research"[Title/Abstract:~3] OR "participatory researches"[Title/Abstract:~3] OR "participatory researched"[Title/Abstract:~3] OR "participatory researching"[Title/Abstract:~3] OR "participation research"[Title/Abstract:~3] OR "participation researches"[Title/Abstract:~3] OR "participation researched"[Title/Abstract:~3] OR "participation researching"[Title/Abstract:~3] OR "participations research"[Title/Abstract:~3] OR "participations researches"[Title/Abstract:~3] OR "participations researched"[Title/Abstract:~3] OR "participations researching"[Title/Abstract:~3] OR "community research"[Title/Abstract:~3] OR "community researches"[Title/Abstract:~3] OR "community researched"[Title/Abstract:~3] OR "community researching"[Title/Abstract:~3] OR "communities research"[Title/Abstract:~3] OR "communities researches"[Title/Abstract:~3] OR "communities researched"[Title/Abstract:~3] OR "communities researching"[Title/Abstract:~3] OR "community participate"[Title/Abstract:~3] OR "Community Participation"[Title/Abstract:~3] OR "community participations"[Title/Abstract:~3] OR "community participates"[Title/Abstract:~3] OR "community participated"[Title/Abstract:~3] OR "community participatory"[Title/Abstract:~3] OR "communities

participate"[Title/Abstract:~3] OR "communities participation"[Title/Abstract:~3] OR "communities participations"[Title/Abstract:~3] OR "communities participates"[Title/Abstract:~3] OR "communities participated"[Title/Abstract:~3] OR "communities participatory"[Title/Abstract:~3] OR "community action"[Title/Abstract:~3] OR "community actions"[Title/Abstract:~3] OR "community act"[Title/Abstract:~3] OR "community acts"[Title/Abstract:~3] OR "community acted"[Title/Abstract:~3] OR "community acting"[Title/Abstract:~3] OR "communities action"[Title/Abstract:~3] OR "communities actions"[Title/Abstract:~3] OR "communities act"[Title/Abstract:~3] OR "communities acts"[Title/Abstract:~3] OR "communities acted"[Title/Abstract:~3] OR "communities acting"[Title/Abstract:~3] OR "community engage"[Title/Abstract:~3] OR "community engages"[Title/Abstract:~3] OR "community engaged"[Title/Abstract:~3] OR "community engagement"[Title/Abstract:~3] OR "community engagements"[Title/Abstract:~3] OR "community engaging"[Title/Abstract:~3] OR "communities engage"[Title/Abstract:~3] OR "communities engages"[Title/Abstract:~3] OR "communities engaged"[Title/Abstract:~3] OR "communities engagement"[Title/Abstract:~3] OR "communities engagements"[Title/Abstract:~3] OR "communities engaging"[Title/Abstract:~3] OR "community partner"[Title/Abstract:~3] OR "community partners"[Title/Abstract:~3] OR "community partnered"[Title/Abstract:~3] OR "community partnership"[Title/Abstract:~3] OR "community partnerships"[Title/Abstract:~3] OR "community partnering"[Title/Abstract:~3] OR "communities partner"[Title/Abstract:~3] OR "communities partners"[Title/Abstract:~3] OR "communities partnered"[Title/Abstract:~3] OR "communities partnership"[Title/Abstract:~3] OR "communities partnerships"[Title/Abstract:~3] OR "communities partnering"[Title/Abstract:~3] OR "community coalition"[Title/Abstract:~3] OR "community coalitions"[Title/Abstract:~3] OR "communities coalition"[Title/Abstract:~3] OR "communities coalitions"[Title/Abstract:~3] OR "community involve"[Title/Abstract:~3] OR "community involves"[Title/Abstract:~3] OR "community involved"[Title/Abstract:~3] OR "community involving"[Title/Abstract:~3] OR "community involvement"[Title/Abstract:~3] OR "community involvements"[Title/Abstract:~3] OR "communities involve"[Title/Abstract:~3] OR "communities involves"[Title/Abstract:~3] OR "communities involved"[Title/Abstract:~3] OR "communities involving"[Title/Abstract:~3] OR "communities involvement"[Title/Abstract:~3] OR "communities involvements"[Title/Abstract:~3] OR "community contribute"[Title/Abstract:~3] OR "community contributes"[Title/Abstract:~3] OR "community contributed"[Title/Abstract:~3] OR "community contribution"[Title/Abstract:~3] OR "community contributions"[Title/Abstract:~3] OR "community contributing"[Title/Abstract:~3] OR "communities contribute"[Title/Abstract:~3] OR "communities contributes"[Title/Abstract:~3] OR "communities

contributed"[Title/Abstract:~3] OR "communities contribution"[Title/Abstract:~3] OR "communities contributions"[Title/Abstract:~3] OR "communities contributing"[Title/Abstract:~3] OR "community cooperate"[Title/Abstract:~3] OR "community cooperates"[Title/Abstract:~3] OR "community cooperated"[Title/Abstract:~3] OR "community cooperating"[Title/Abstract:~3] OR "community cooperation"[Title/Abstract:~3] OR "communities cooperate"[Title/Abstract:~3] OR "communities cooperates"[Title/Abstract:~3] OR "communities cooperated"[Title/Abstract:~3] OR "communities cooperating"[Title/Abstract:~3] OR "communities cooperation"[Title/Abstract:~3] OR "community inform"[Title/Abstract:~3] OR "community informs"[Title/Abstract:~3] OR "community informed"[Title/Abstract:~3] OR "community informing"[Title/Abstract:~3] OR "community information"[Title/Abstract:~3] OR "communities inform"[Title/Abstract:~3] OR "communities informs"[Title/Abstract:~3] OR "communities informed"[Title/Abstract:~3] OR "communities informing"[Title/Abstract:~3] OR "communities information"[Title/Abstract:~3] OR "community consult"[Title/Abstract:~3] OR "community consults"[Title/Abstract:~3] OR "community consulted"[Title/Abstract:~3] OR "community consulting"[Title/Abstract:~3] OR "community consultation"[Title/Abstract:~3] OR "community consultations"[Title/Abstract:~3] OR "community consultant"[Title/Abstract:~3] OR "community consultants"[Title/Abstract:~3] OR "communities consult"[Title/Abstract:~3] OR "communities consults"[Title/Abstract:~3] OR "communities consulted"[Title/Abstract:~3] OR "communities consulting"[Title/Abstract:~3] OR "communities consultation"[Title/Abstract:~3] OR "communities consultations"[Title/Abstract:~3] OR "communities consultant"[Title/Abstract:~3] OR "communities consultants"[Title/Abstract:~3] OR "community empower"[Title/Abstract:~3] OR "community empowers"[Title/Abstract:~3] OR "community empowerment"[Title/Abstract:~3] OR "community empowered"[Title/Abstract:~3] OR "community empowering"[Title/Abstract:~3] OR "community empowerments"[Title/Abstract:~3] OR "communities empower"[Title/Abstract:~3] OR "communities empowers"[Title/Abstract:~3] OR "communities empowerment"[Title/Abstract:~3] OR "communities empowered"[Title/Abstract:~3] OR "communities empowering"[Title/Abstract:~3] OR "communities empowerments"[Title/Abstract:~3] OR "community effort"[Title/Abstract:~3] OR "community efforts"[Title/Abstract:~3] OR "community efforted"[Title/Abstract:~3] OR "communities effort"[Title/Abstract:~3] OR "communities efforts"[Title/Abstract:~3] OR "communities efforted"[Title/Abstract:~3] OR "community base"[Title/Abstract:~3] OR "community based"[Title/Abstract:~3] OR "community bases"[Title/Abstract:~3] OR "community basing"[Title/Abstract:~3] OR "community basis"[Title/Abstract:~3] OR "communities base"[Title/Abstract:~3] OR "community bases"[Title/Abstract:~3] OR

	"communities based"[Title/Abstract:~3] OR "communities basing"[Title/Abstract:~3] OR "communities basis"[Title/Abstract:~3]<br><br>**Legend**<br>*: truncation of word<br>~n: words terms are within ‘n’ number of words within each other, in either direction<br>Mesh: Medical subject heading
The Cochrane Library	MeSH descriptor: [Child] explode all trees OR MeSH descriptor: [Adolescent] explode all trees OR MeSH descriptor: [Child, Preschool] explode all trees or (pediatr* or paediatr* or child* or toddler* or preschool* or preteen* or teen* or adolesce* or youth* or juvenile* or kid*):ti,ab,kw<br><br>AND<br><br>MeSH descriptor: [Pediatric Obesity] explode all trees OR MeSH descriptor: [Obesity] explode all trees OR MeSH descriptor: [Overweight] explode all trees OR (overweigh* or obes*):ti,ab,kw<br><br>AND<br><br>MeSH descriptor: [Community Participation] explode all trees OR MeSH descriptor: [Community-Based Participatory Research] explode all trees OR (CBPR or (participat* NEAR/3 research*) or ((communit*) NEAR/3 (participat* or research* or act* or engag* or partner* or coalition* or involv* or contribut* or cooperat* or inform* or consult* or empower* or effort* or base* OR basing* OR basis*))):ti,ab,kw<br><br>**Legend**<br>*: word truncation<br>MeSH: Medical Subject Heading<br>NEAR/n: terms are within ‘n’ number of words within each other, in either direction<br>ti,ab,kw: title, abstract, keyword

Cinahl (EBSCO)	MH "Child+" OR MH "Adolescence+" OR TI (pediatr* or paediatr* or child* or toddler* or preschool* or preteen* or teen* or adolesce* or youth* or juvenile* or kid*) OR AB (pediatr* or paediatr* or child* or toddler* or preschool* or preteen* or teen* or adolesce* or youth* or juvenile* or kid*)<br><br>AND<br><br>MH "Pediatric Obesity" OR MH “Obesity+” OR TI (overweigh* or obes*) OR AB (overweight* or obes*)<br><br>AND<br><br>MH "Community Role" OR MH "Stakeholder Participation" OR MH "Consumer Participation" OR MH "Action Research" OR TI (“CBPR” or (participat* N3 research*) or (communit* N3 (participat* or research* or act* or engag* or partner* or coalition* or involv* or contribut* or cooperat* or inform* or consult* or empower* or effort* or base* or basing* or basis*))) OR AB (“CBPR” or (participat* N3 research*) or (communit* N3 (participat* or research* or act* or engag* or partner* or coalition* or involv* or contribut* or cooperat* or inform* or consult* or empower* or effort* or base* or basing* or basis*)))<br><br>**<u>Legend</u>**<br>*: truncation of word<br>+: explode<br>AB: abstract<br>MH: major subject heading<br>Nn: the terms are ‘n’ number of words within each other, in either direction<br>TI: title
Psycinfo (Ovid)	exp Nursery School Students/ OR exp Preschool Students/ OR exp Kindergarten Students/ OR exp Elementary School Students/ OR exp Junior High School Students/ OR exp High School Students/ OR (pediatr* or paediatr* or child* or toddler* or preschool* or preteen* or teen* or adolesce* or youth* or juvenile* or kid*).ab,ti,tw<br><br>AND<br><br>exp Obesity/ OR exp Overweight/ OR (overweigh* or obes*).ab,ti,tw.

	AND<br><br>exp Community Involvement/ OR exp Action Research/ OR (“CBPR” or (participat* adj3 research*) or (communit* adj3 (participat* or research* or act* or engag* or partner* or coalition* or involv* or contribut* or cooperat* or inform* or consult* or empower* or effort* or base* or basing* or basis*))).ab,ti,tw.<br><br>**<u>Legend</u>**<br>*: truncation of word<br>ab: abstract<br>adj/n: the terms are ‘n’ number of words within each other, in either direction<br>Exp: explode<br>ti: title<br>tw: text word
ProQuest Dissertations & Theses Global	subject(Children & youth) OR subject(Toddlers) OR subject(Preschool children) OR subject(Teenagers) OR noft(pediatr* OR paediatr* OR child* OR toddler* OR preschool* OR preteen* OR teen* OR adolesce* OR youth* OR juvenile* OR kid*)<br><br>AND<br><br>subject(Overweight) OR subject(Obesity) OR noft(overweigh* OR obes*)<br><br>AND<br><br>subject(Participatory research) OR subject(Citizen participation) OR subject(Community action) OR noft(“CBPR” OR (participat* NEAR/3 research*) OR (communit* NEAR/3 (participat* OR research* OR act* OR engag* OR partner* OR coalition* OR involv* OR contribut* OR cooperat* OR inform* OR consult* OR empower* OR effort* OR base* OR basing* OR basis*)))<br><br>**<u>Legend</u>**<br>*: truncation of word

	NEAR/n: the terms are 'n' number of words within each other, in either direction<br>noft: not in full text
Web of science (Thomson Reuters)	TS=(child* OR adolesce* OR juvenile* OR pediatr* OR paediatr* OR toddler* OR preschool* OR preteen* OR teen* OR youth* OR kid*)<br><br>AND<br><br>TS=(overweigh* OR obes*)<br><br>AND<br><br>TS=("CBPR" OR (participat* NEAR/3 research*) OR (communit* NEAR/3 (participat* OR research* OR act* OR engag* OR partner* OR coalition* OR involv* OR contribut* OR cooperat* OR inform* OR consult* OR empower* OR effort* OR base* OR basing* OR basis*)))<br><br>**Legend**<br>*: truncation of word<br>TS: Topic (Searches title, abstract, author keywords, and Keywords Plus)
Scopus (Elsevier)	TITLE-ABS-KEY (child* OR adolesce* OR juvenile* OR pediatr* OR paediatr* OR toddler* OR preschool* OR preteen* OR youth* OR kid*)<br><br>AND<br><br>TITLE-ABS-KEY (overweight* OR obes*)<br><br>AND<br><br>TITLE-ABS-KEY ("cbpr" OR (participat* w/3 research*) OR (communit* w/3 (participat* OR research* OR act* OR engag* OR partner* OR coalition* OR involv* OR contribut* OR cooperat* OR inform* OR consult* OR empower* OR effort* OR base* OR basing* OR basis*)))

Legend

*: truncation of word

ABS: abstract

KEY: keyword

W/n: the terms are ‘n’ number of words within each other, in either direction

Table S2. Table of included studies' characteristics

Study ID (Country)	Research Design/ Study Arms	Participant number/ Age mean (SD) or mean (range) or range	Community engagement:<br><br>1. Planning<br>2. Design<br>3. Implementation<br>4. Data collection<br>5. Results analysis/dissemination	Intervention content<br><br>(intervention change: behavioral/ environmental change/both)	Setting/ Personnel<br><br>(mode of delivery: calls/in-person/newsletter/online)	Intervention overall duration<br><br>(follow up from post-intervention)	Outcomes:<br>1. BMI-z<br>2. Fruits<br>3.Vegetables<br>4. SSB<br>5. MVPA<br>6. SST<br>7. EGT	Attrition rate	Any framework?<br><br>ITT?
Adab et al., 2018 (United Kingdom)	2 arm Cluster RCT:<br>1. WAVES (n = 1134)<br>2. Usual care (n = 1328)	T: Year 2 primary school children<br>Age: 6-7 years old	1. Yes<br>2. Yes<br>3. Yes<br>4. No<br>5. No	-Health behavior skills<br>-Physical activity<br><br>(behavioral change)	School-based/ class teachers, school staff, sporting institution community coaches and staff<br><br>(in-person)	1 year<br><br>(18 months)	1. Yes<br>2. No<br>3. No<br>4. No<br>5. Yes<br>6. No<br>7. No	Post test:<br>I: 13.9%<br>C: 10.8%<br>T: 12.3%<br><br>Follow up (30 months):<br>I: 23.2%<br>C: 17.7%<br>T: 20.3%<br>Follow up (39 months):<br>I: 67.3%<br>C: 65.3%<br>T: 66.3%	Yes<br><br>Yes
Black et al., 2010 (United States)	2 arm RCT:<br>1. Challenge (n = 121)<br>2. Control (n = 114)	I: 62 girls, 59 boys<br>Age: 13.3 years (1.0)<br>C: 54 girls, 60 boys<br>Age: 13.3 years (1.0)	1. No<br>2. Yes<br>3. Yes<br>4. No<br>5. No	-Goal setting<br>-Healthy Eating<br>-Mentorship<br>-Participatory learning<br>-Physical activity<br>-Progress tracking<br><br>(behavioral change)	Community- and home-based/ college-enrolled mentors<br><br>(in-person)	10 months<br><br>(14 months)	1. Yes<br>2. Yes<br>3. Yes<br>4. Yes<br>5. No<br>6. No<br>7. No	Post test:<br>I: 24.8%<br>C: 18.4%<br>T: 21.7%<br><br>Follow up:<br>I: 26.4%<br>C: 21.1%<br>T: 23.8%	Yes<br><br>Yes
Buch-Andersen	2 arm CCT:<br>1. Danish Sundhed og	I: 105 girls, 96 boys	1. Yes<br>2. Yes<br>3. Yes	-Capacity building<br>-Healthy eating practices	Community- and school-based/ after-school centers, childcare centers, civil	19 months<br><br>(no follow up)	1. Yes<br>2. No<br>3. No	Baseline:<br>I: 46.3%<br>C: 48.8%	Yes<br><br>Yes

et al., 2021 (Denmark)	Lokasamfund (n = 238)<br>2. Usual care (n = 214)	Age: 6.51 years (1.49)<br>C: 90 girls, 76 boys<br>Age: 6.21 years (1.80)	4. No<br>5. No	-Physical activity<br><br>(both)	society organizations, local mass media, local stakeholders, primary schools, resource persons with various expertise, supermarkets<br><br>(in-person)		4. No<br>5. No<br>6. No<br>7. No	T: 47.5%<br><br>Post test:<br>I: 24.5%<br>C:21.7%<br>T: 23.2%	
Crespo et al., 2012 (United States)	4 arm Cluster RCT<br>1. Community only [Comm] (n = 218)<br>2. Family only [F] (n = 198)<br>3. Community and Family [Comm+F] (n = 165)<br>4. Control (n = 227)	T: kindergarten to Grade 2 elementary school children	1. No<br>2. Yes<br>3. Yes<br>4. No<br>5. No	-Active play<br>-Environmental change<br>-Healthy eating<br>-Screen time<br><br>(environmental change)	Community- and home-based/ promotoras<br><br>(calls, in-person)	1 year<br><br>(1 year, 2 years)	1. Yes<br>2. No<br>3. No<br>4. Yes<br>5. No<br>6. No<br>7. No	Post test:<br>Comm: 9.3%<br>F: 13.9%<br>Comm+F: 11.0%<br>C: 8.1%<br>T: 10.4%<br><br>Follow up (1 year):<br>Comm: 28.7%<br>F: 27.8%<br>Comm+F: 27.0%<br>C: 23.3%<br>T: 26.6%<br>Follow up (2 years):<br>Comm: 40.7%<br>F: 50.5%<br>Comm+F: 49.1%<br>C: 39.9%<br>T: 44.5%	Yes<br><br>No
Davis et al., 2016 (United States)	2 arm RCT:<br>1. CHILE (n = 214)<br>2. Usual care (n = 229)	T: 4-6 years old children	1. Yes<br>2. Yes<br>3. Yes<br>4. No<br>5. No	-Availability and visibility of healthier food options in grocery stores<br>-Family engagement<br>-Food service<br>-Local healthcare provider engagement	Community- and school-based/ grocery stores, Head Start staff, healthcare providers, schools	2 years<br><br>(no follow up)	1. Yes<br>2. No<br>3. No<br>4. No<br>5. No<br>6. No<br>7. No	(attrition not stated)	Yes<br><br>Yes

				-Nutrition<br>-Physical activity<br><br>(both)					
Economos et al., 2013 (United States)	2 arm CCT:<br>1. Shape Up Somerville (n = 647)<br>2. Control (n = 1074)	T: Grade 1-3 children	1. Yes<br>2. Yes<br>3. Yes<br>4. No<br>5. No	-Access and availability of physical activity options and healthy food<br><br>(both)	Community-, home- and school-based/ before-and after-school programs, city departments, food service providers, healthcare providers, media, parents, policy makers, restaurants, teachers<br><br>(in-person)	2 years<br><br>(no follow up)	1. Yes<br>2. No<br>3. No<br>4. No<br>5. No<br>6. No<br>7. No	Post test:<br>I: 27.3%<br>C: 22.1%<br>T: 23.9%	Yes<br><br>Yes
Elinder et al., 2012 (Sweden)	2 arm CCT:<br>1. SCIP-school programme (n = 328)<br>2. Usual care (n = 478)	T: Grade 2, 4, and 7 children	1. Yes<br>2. Yes<br>3. Yes<br>4. Yes<br>5. No	-Capacity building<br>-Eating habits<br>-Physical activity<br>-Self-esteem<br><br>(both)	School-based/ local school health teams, school staff<br><br>(in-person)	2 years<br><br>(no follow up)	1. Yes<br>2. Yes<br>3. Yes<br>4. Yes<br>5. No<br>6. Yes<br>7. No	Post test:<br>BMI-z:<br>I: 5.5%<br>C: 12.6%<br>T: 9.7%<br><br>Behaviour:<br>I: 14%<br>C: 14%<br>T: 14%	Yes<br><br>Yes
Folta et al., 2013‡ (United States)	2 arm CCT:<br>1. Shape Up Somerville (n = 647)<br>2. Control (n = 1074)	T: Grade 1-3 children	1. Yes<br>2. Yes<br>3. Yes<br>4. No<br>5. No	-Access and availability of physical activity options and healthy food<br><br>(both)	Community-, home- and school-based/ before-and after-school programs, city departments, food service providers, healthcare providers, media, parents, policy makers, restaurants, teachers<br><br>(in-person)	2 years<br><br>(no follow up)	1. No<br>2. No<br>3. No<br>4. Yes<br>5. No<br>6. Yes<br>7. No	Post test:<br>Behaviour:<br>I: 65.9%<br>C: 45.5%<br>T: 52.4%	Yes<br><br>Yes
Fotu et al., 2011 (Tonga)	2 arm CCT:<br>1. Ma'alahi Youth Project (n = 2610)<br>2. Usual care (n = 2182)	I: 441 girls, 374 boys<br>Age: 14.4 years (2.0)<br>C: 526 girls, 371 boys<br>Age: 15.2 years (1.8)	1. Yes<br>2. Yes<br>3. Yes<br>4. No<br>5. No	-Capacity building<br>-Education<br>-Local fruit and vegetable promotion<br>-Physical activity<br>-Social marketing	Community-, policy- and school-based/ churches, community leaders, government, mass media, schools, student ambassadors, teachers, villages, youth groups,<br><br>(in-person)	2.5 years<br><br>(no follow up)	1. Yes<br>2. No<br>3. No<br>4. Yes<br>5. No<br>6. Yes<br>7. Yes	Post test:<br>I: 24.7%<br>C: 35.7%<br>T: 30.9%	Yes<br><br>No

				(environmental change)					
Fulkerson et al., 2022[⊕] (United States)	2 arm RCT:<br>1. New Ulm at Healthy Offerings via the Mealtime Environment (n = 58)<br>2. Wait-list control (n = 56)	I: 30 girls, 26 boys<br>Age: 9.0 years (1.0)<br>C: 30 girls, 16 boys<br>Age: 9.0 years (1.1)	1. Yes<br>2. Yes<br>3. Yes<br>4. No<br>5. No	-Capacity building<br>-Cooking together as a family<br>-Healthful home food environment<br>-Positive family activities<br>-Screen time<br>-Snacks and family meals<br><br>(both)	Community-based/ health educator, registered dietician, students<br><br>(calls, in-person)	7 months<br><br>(no follow up)	1. Yes<br>2. Yes<br>3. Yes<br>4. No<br>5. Yes<br>6. No<br>7. No	Post test:<br>I: 3.6%<br>C: 4.3%<br>T: 3.9%	Yes<br><br>Yes
Gómez et al., 2018 (Spain)	2 arm Cluster RCT:<br>1. Thao-Child Health Program (n = 1041)<br>2. Control (n = 1209)	I: 445 girls, 529 boys<br>Age: 10.2 years (0.64)<br>C: 556 girls, 556 boys<br>Age: 10.1 years (0.60)	1. No<br>2. Yes<br>3. Yes<br>4. No<br>5. No	-Capacity building<br>-Dietary intake<br>-Healthy lifestyle<br>-Physical activity<br><br>(behavioral change)	Community- and school-based/ associations, companies, health professionals, kindergarten, local producers and shop owners, media, schools, sports clubs<br><br>(in-person)	2 years<br><br>(no follow up)	1. Yes<br>2. No<br>3. No<br>4. No<br>5. No<br>6. No<br>7. No	Baseline:<br>I: 10.4%<br>C: 13.3%<br>T: 12.0%<br><br>Post test:<br>I: 6.4%<br>C: 8.0%<br>T: 7.3%	Yes<br><br>Yes
Kattelmann et al., 2019[‡] (United States)	2 arm RCT:<br>1. iCook 4-H (n = 151)<br>2. Control (n = 77)	T: 125 girls, 103 boys<br>Age: 9.35 years (0.67)	1. Yes<br>2. Yes<br>3. Yes<br>4. Yes<br>5. No	-Develop cooking skills<br>-Enhance family mealtime<br>-Physical activity<br><br>(behavioral change)	Community-based/ 4-H staff, community paraprofessionals, extension educators, high school students, nutrition and nursing students, nutrition professionals, paraprofessionals<br><br>(in-person)	2 years<br><br>(no follow up)	1. No<br>2. No<br>3. No<br>4. No<br>5. Yes<br>6. No<br>7. No	Post test:<br>I: 40.4%<br>C: 54.5%<br>T: 45.2%	Yes<br><br>Yes
Kremer et al., 2011 (Fiji)	2 arm CCT:<br>1. Healthy Youth, Healthy Communities (n = 23530)<br>2. Usual care (n = 6255)	T: Secondary school students<br>Age: 13-18 years old	1. Yes<br>2. Yes<br>3. Yes<br>4. No<br>5. No	-Community capacity building<br>-Healthy eating<br>-Physical activity<br><br>(environmental change)	Community- and school-based / church groups, road traffic control officers, schools, teachers<br><br>(in-person)	2 years<br><br>(no follow up)	1. Yes<br>2. Yes<br>3. Yes<br>4. Yes<br>5. No<br>6. Yes<br>7. No	Post test:<br>I: 67.1%<br>C: 54.7%<br>T: 59.3%	Yes<br><br>No
Malakellis et al., 2017	2 arm CCT:<br>1. Australian Capital	T: Secondary school students	1. Yes<br>2. Yes<br>3. Yes	-Active transport<br>-Capacity building<br>-Healthy eating	Community- and school-based/ local food producers, non-government and non-	3 years<br><br>(no follow up)	1. Yes<br>2. Yes<br>3. Yes	Post test:<br>I: 20.5%<br>C: 37.7%	Yes<br><br>No

(Australia)	Territory 'It's Your Move!' (n = 891)<br>2. Usual care (n = 666)	Age: 12-16 years	4. No<br>5. No	-Healthy food policies<br>-Physical activity<br><br>(environmental change)	profit organization, school project officers, student ambassadors<br><br>(in-person)		4. Yes<br>5. No<br>6. Yes<br>7. No	T: 25.5%	
Millar et al., 2011 (Australia)	2 arm CCT:<br>1. It's Your Move! (n = 3505)<br>2. Usual care (n = 2508)	T: Secondary school students<br>Age: 14.6 years (1.42)	1. Yes<br>2. Yes<br>3. Yes<br>4. No<br>5. No	-Capacity building<br>-Healthy eating<br>-Physical activity<br><br>(environmental change)	Community- and school-based/ schools, school project officers, student ambassadors, teachers, walking groups<br><br>(in-person)	3 years<br><br>(no follow up)	1. Yes<br>2. Yes<br>3. Yes<br>4. Yes<br>5. No<br>6. Yes<br>7. No	Post test:<br>I: 31.3%<br>C: 34.5%<br>T: 32.4%	Yes<br><br>No
Peña et al.,2021 (United States)	2 arm Cluster RCT:<br>1. Juntos Santiago (n = 2299)<br>2. Usual care + access to educational leaflets and videos (n = 1573)	I: 670 girls,941 boys<br>Age: 11.08 years (0.75)<br>C: 252 girls, 45y boys<br>Age: 11.15 years (0.79)	1. No<br>2. Yes<br>3. Yes<br>4. No<br>5. No	-Gamification<br>-Healthy snacks<br>-Physical activity<br>-Reward system<br><br>(both)	School-based/ trained monitors<br><br>(in-person, online)	7 months<br><br>(no follow up)	1. Yes<br>2. No<br>3. No<br>4. No<br>5. No<br>6. No<br>7. No	Post test:<br>I: 7.8%<br>C: 7.1%<br>T: 7.6%	Yes<br><br>Yes
Sanigorski et al., 2008 (Australia)	2 arm CCT:<br>1. Be Active Eat Well (n = 1001)<br>2. Usual care (n = 1183)	I: 447 girls, 386 boys<br>Age: 11.13 years (2.27)<br>C: 478 girls, 496 boys<br>Age: 10.31 years (2.14)	1. Yes<br>2. Yes<br>3. Yes<br>4. No<br>5. No	-Capacity building<br>-Nutrition<br>-Physical activity<br>-Screen time<br><br>(both)	Community-, policy- and school-based/ dietician, government, schools, media, teachers<br><br>(in-person)	3 years<br><br>(no follow up)	1. Yes<br>2. No<br>3. No<br>4. No<br>5. No<br>6. No<br>7. No	Post test:<br>I: 16.8%<br>C: 17.7%<br>T: 13.4%<br><br>Follow up:<br>I: 24.7%<br>C: 22.1%<br>T: 23.5%	Yes<br><br>No
Scherr et al., 2017 (United States)	2 arm Cluster RCT:<br>1. Shaping Healthy Choices Program (n = 198)<br>2. Usual care (n = 245)	I: 94 girls, 104 boys<br>Age: 9.63 years (0.37)<br>C: 117 girls,128 boys<br>Age: 9.52 years (0.36)	1. Yes<br>2. Yes<br>3. Yes<br>4. No<br>5. No	-Dietary intake<br>-Physical activity<br><br>(both)	School-based/ academic researchers, newsletter, nutrition educator, school<br><br>(in-person, newsletter)	4 years<br><br>(no follow up)	1. Yes<br>2. Yes<br>3. Yes<br>4. No<br>5. No<br>6. No<br>7. No	Post test:<br>I: 2.4%<br>C: 1.3%<br>T: 1.8%	Yes<br><br>No

Taylor et al., 2007 (New Zealand)	2 arm CCT:<br>1. APPLE Project (n = 279)<br>2. Control (n = 234)	I: 112 girls, 138 boys<br>Age: 7.7 years (1.8)<br>C: 111 girls, 108 boys<br>Age: 7.7 years (1.6)	1. Yes<br>2. Yes<br>3. Yes<br>4. No<br>5. No	-Healthy eating<br>-Noncurricular physical activity<br><br>(both)	Community- and school-based/ community activity coordinators, schools, teachers<br><br>(in-person)	2 years<br><br>(no follow up)	1. Yes<br>2. Yes<br>3. Yes<br>4. Yes<br>5. No<br>6. No<br>7. No	Post test:<br>I: 45.9%<br>C: 41.5%<br>T: 43.9%	Yes<br><br>No
Tomayko et al., 2019 (United States)	2 arm RCT:<br>1. Wellness journey (n = 225)<br>2. Safety journey (n = 225)	I: 115 girls, 110 boys<br>Age: 45.9months (12.8)<br>C: 111 girls, 114 boys<br>Age: 44.1 months (13.2)	1. No<br>2. Yes<br>3. No<br>4. No<br>5. No	-Healthy eating habits<br>-Physical activity<br>-Screen time<br>-Sleep habits<br><br>(behavioral change)	Home-based/ site coordinators, parents<br><br>(in-person, newsletter, online)	1 year<br><br>(no follow up)	1. Yes<br>2. No<br>3. No<br>4. Yes<br>5. No<br>6. No<br>7. No	(attrition not stated)	Yes<br><br>No
White et al., 2019 (United States)	2 arm RCT:<br>1. iCook 4-H (n = 151)<br>2. Control (n = 77)	T: 125 girls, 103 boys<br>Age: 9.35 years (0.67)	1. Yes<br>2. Yes<br>3. Yes<br>4. Yes<br>5. No	-Develop cooking skills<br>-Enhance family mealtime<br>-Physical activity<br><br>(behavioral change)	Community-based/ 4-H staff, community paraprofessionals, extension educators, high school students, nutrition and nursing students, nutrition professionals, paraprofessionals<br><br>(in-person)	2 years<br><br>(no follow up)	1. Yes<br>2. No<br>3. No<br>4. No<br>5. No<br>6. No<br>7. No	Post test:<br>I: 40.4%<br>C: 54.5%<br>T: 45.2%	Yes<br><br>Yes
Wong et al., 2016 (United States)	2 arm CCT:<br>1. Heathy Kids-Houston Program (n = 524)<br>2. Usual care (n = 353)	I: 265 girls, 259 boys<br>Age: 10.30 years (0.04)<br>C: 156 girls, 197 boys<br>Age: 10.22 years (0.05)	1. No<br>2. Yes<br>3. Yes<br>4. No<br>5. No	-Dietary intake<br>-Healthy lifestyle<br>-Physical activity<br><br>(behavioral change)	Community-based/ clinical pediatric psychologist, community centers, government, health fitness professional, nutritional epidemiologist, school, transport authorities<br><br>(in-person)	18 weeks<br><br>(no follow up)	1. Yes<br>2. No<br>3. No<br>4. No<br>5. Yes<br>6. No<br>7. No	Post test:<br>I: 45.0%<br>C: 47.0%<br>T: 45.8%	No<br><br>No

Legend: ANGELO = Analysis Grid for Elements Linked to Obesity; AR = action research methodology; BMI = body mass index; BMI-z = standardized BMI score; C = control; CBPR = community-based participatory research; CCT = controlled clinical trial; Comm = Community-only intervention; Comm+F = Family-plus-Community intervention; EGT = electronic game time; F&V = fruits and vegetables; F = Family-only intervention; I = intervention; ITT = intent-to-treat; MVPA = moderate-to-vigorous activity; N = No; NA = not applicable; RCT = randomized controlled trial; SD = standard deviation; SSB = sugar-sweetened beverages; SST = small screen time [i.e., television (TV), video, digital versatile discs (DVD)]; T = total; TST = total screen time; Y = Yes; ‡ = reported secondary outcomes for main study only; ⊕ = secondary outcomes reported at clinicaltrials.gov

Table S3. GRADE summary of evidence table

Certainty assessment							№ of patients		Effect		Certainty	Importance
№ of studies	Study design	Risk of bias	Inconsistency	Indirectness	Imprecision	Other considerations	Universal community engagement interventions	Comparison	Relative (95% CI)	Absolute (95% CI)		
**BMI-z score**												
12	randomised trials	very serious[a]	not serious[b]	serious[d]	not serious[e]	none[j]	6918	5381	-	MD **0.02 lower** (0.07 lower to 0.03 higher)	⨁◯◯◯ Very low	IMPORTANT
**Fruit intake (dichotomous)**												
3	randomised trials	very serious[a]	not serious[b]	serious[d]	not serious[f]	none	1252/2641 (47.4%)	1191/2995 (39.8%)	**RR 0.94** (0.87 to 1.02)	**24 fewer per 1,000** (from 52 fewer to 8 more)	⨁◯◯◯ Very low	IMPORTANT
**Fruit intake (continuous)**												

Certainty assessment							№ of patients		Effect		Certainty	Importance
**№ of studies**	**Study design**	**Risk of bias**	**Inconsistency**	**Indirectness**	**Imprecision**	**Other considerations**	**Universal community engagement interventions**	**Comparison**	**Relative (95% CI)**	**Absolute (95% CI)**		
3	randomised trials	very serious[a]	not serious[b]	serious[d]	not serious[g]	none	254	223	-	SMD **0.03 SD lower** (0.24 lower to 0.17 higher)	⨁◯◯◯ Very low	IMPORTANT
**Vegetable intake (dichotomous)**												
3	randomised trials	very serious[a]	not serious[b]	serious[d]	not serious[h]	none	1486/2641 (56.3%)	1828/2995 (61.0%)	**RR 0.92** (0.89 to 0.96)	**49 fewer per 1,000** (from 67 fewer to 24 fewer)	⨁◯◯◯ Very low	IMPORTANT
**Vegetable intake (continuous)**												
3	randomised trials	very serious[a]	not serious[b]	serious[d]	not serious[g]	none	254	223	-	SMD **0.02 SD lower** (0.23 lower to 0.19 higher)	⨁◯◯◯ Very low	IMPORTANT

Certainty assessment							№ of patients		Effect		Certainty	Importance
№ of studies	Study design	Risk of bias	Inconsistency	Indirectness	Imprecision	Other considerations	Universal community engagement interventions	Comparison	Relative (95% CI)	Absolute (95% CI)		
**Sugar-sweetened beverages (dichotomous)**												
4	randomised trials	very serious[a]	very serious[c]	serious[d]	not serious[f]	none	1843/2868 (64.3%)	2515/3432 (73.3%)	**RR 0.93** (0.82 to1.05)	**51 fewer per 1,000** (from 132 fewer to 37 more)	⊕◯◯◯ Very low	IMPORTANT
**Sugar-sweetened beverages (continuous)**												
3	randomised trials	very serious[a]	not serious[b]	serious[d]	not serious[g]	none	671	563	-	SMD **0.25 SD lower** (0.38 lower to 0.13 lower)	⊕◯◯◯ Very low	IMPORTANT
**Moderate-to-vigorous physical activity (continuous)**												
4	randomised trials	very serious[a]	not serious[b]	serious[d]	not serious[i]	none	856	795	-	SMD **0 SD lower** (0.10 lower to 0.1 higher)	⊕⊕◯◯ Low	IMPORTANT

Certainty assessment							№ of patients		Effect		Certainty	Importance
№ of studies	Study design	Risk of bias	Inconsistency	Indirectness	Imprecision	Other considerations	Universal community engagement interventions	Comparison	Relative (95% CI)	Absolute (95% CI)		
**Small screen time (dichotomous)**												
4	randomised trials	very serious[a]	very serious[c]	serious[d]	not serious[f]	none	1911/2804 (68.2%)	2547/3432 (74.2%)	**RR 0.95** (0.86 to 1.05)	**37 fewer per 1,000** (from 104 fewer to 37 more)	⨁◯◯◯ Very low	IMPORTANT
**Electronic game time (dichotomous)**												
4	randomised trials	very serious[a]	very serious[c]	serious[d]	not serious[f]	none	1535/2804 (54.7%)	2183/3432 (63.6%)	**RR 0.93** (0.81 to 1.07)	**45 fewer per 1,000** (from 121 fewer to 45 more)	⨁◯◯◯ Very low	IMPORTANT
Legend: **CI** = confidence interval; **MD** = mean difference; **RR -** risk ratio; **SMD** = standardized mean difference												

Explanations

a. Almost all studies have unclear/high overall bias

b. I-square statistics ≤40%, low heterogeneity

c. I-square statistics ≥75%, considerable heterogeneity

d. Universal pediatric populations were from different countries, undergoing different interventions of different durations

e. No studies with small sample size, and only one study with wide CI (upper or lower limit crosses MD of 0.5 in either direction)

f. No studies with small sample size and wide CI (upper or lower limit crosses RR of 0.25 in either direction)

g. No studies with small sample size and wide CI (upper or lower limit crosses SMD of 0.5 in either direction)

h. No studies with small sample size. Only one study with wide CI (upper or lower limit crosses RR of 0.25 in either direction)

i. No studies with small sample size. Only one study with wide CI (upper or lower limit crosses SMD of 0.5 in either direction)

j. Visual inspection of funnel plot indicates no observed pattern of publication bias
