# Supplementary material for: Effectiveness of Universal Community Engagement Childhood Obesity Interventions at Improving Weight-Related and Behavioral Outcomes among Children and Adolescents: A Systematic Review and Meta-Analysis
Source: Nutrients. 2024 Oct 12;16(20):3465. doi: 10.3390/nu16203465 (PMC11510657; doi:10.3390/nu16203465)

Supplementary File S2: Figures S1-S16

Figure S1. Forest plot of BMI-z scores at post-intervention in 12 included studies

Study or Subgroup	Intervention Mean	Intervention SD	Intervention Total	Control Mean	Control SD	Control Total	Weight	Mean difference IV, Random, 95% CI
Black et al., 2010	0.73	1.16	91	0.61	1.14	93	1.9%	0.12 [-0.21 , 0.45]
Crespo et al., 2012 (Community + Home)	0.96	1.06	196	0.99	1.12	69	2.3%	-0.03 [-0.33 , 0.27]
Crespo et al., 2012 (Community only)	0.85	1.09	145	0.99	1.12	68	2.1%	-0.14 [-0.46 , 0.18]
Crespo et al., 2012 (Home only)	0.95	1.13	167	0.99	1.12	68	2.1%	-0.04 [-0.36 , 0.28]
Davis et al., 2016	0.81	1.170299	214	0.8	1.165221	229	4.2%	0.01 [-0.21 , 0.23]
Fotu et al., 2011	1.1	0.9	815	1.1	0.9	897	17.4%	0.00 [-0.09 , 0.09]
Fulkerson et al., 2022	0.69	1	56	1.03	0.89	46	1.6%	-0.34 [-0.71 , 0.03]
Gómez et al., 2018	0.64	1.12	974	0.57	1.15	1112	14.8%	0.07 [-0.03 , 0.17]
Millar et al., 2011	0.5	1.05	1276	0.53	0.95	778	16.7%	-0.03 [-0.12 , 0.06]
Peña et al., 2021	0.92	1.15	1544	1.07	1.19	653	13.0%	-0.15 [-0.26 , -0.04]
Sanigorski et al., 2008	0.54	0.94	833	0.58	0.88	974	17.6%	-0.04 [-0.12 , 0.04]
Scherr et al., 2017	0.53	1.04	229	0.41	1.1	172	4.4%	0.12 [-0.09 , 0.33]
White et al., 2019	0.82	1.043552	90	0.65	0.857832	35	1.7%	0.17 [-0.19 , 0.53]
Wong et al., 2016	1.42	17.819091	288	1.41	14.495282	187	0.0%	0.01 [-2.91 , 2.93]
**Total (95% CI)**			**6918**			**5381**	**100.0%**	**-0.02 [-0.07 , 0.03]**

Heterogeneity: Tau² = 0.00; Chi² = 16.32, df = 13 (P = 0.23); I² = 20%
Test for overall effect: Z = 0.83 (P = 0.40)
Test for subgroup differences: Not applicable

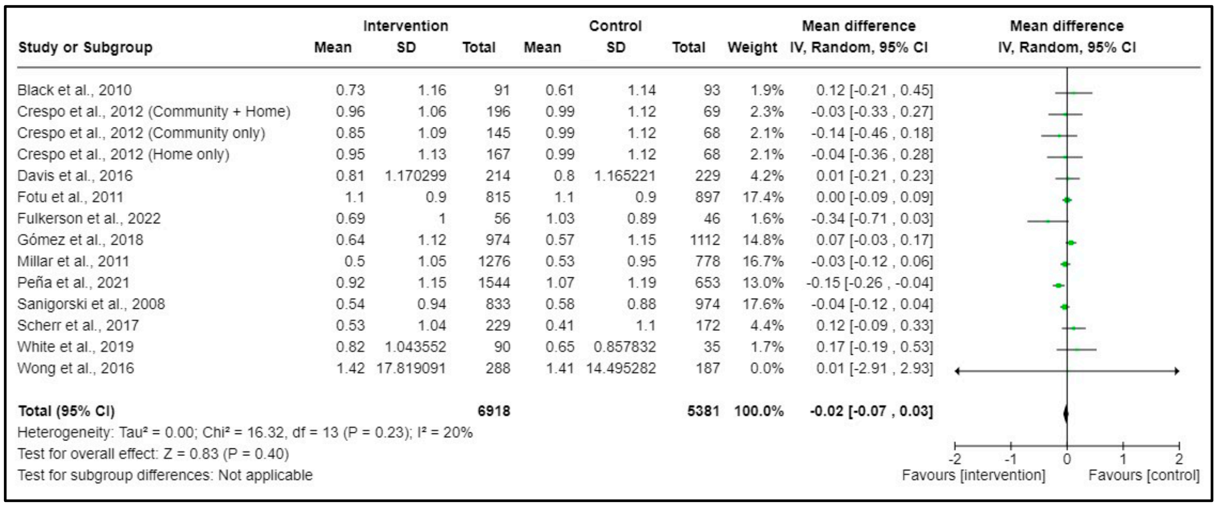

Figure S2. Forest plots of subgroup analyses on BMI-z at post-intervention according to a) number of community engagement research stages, and b) type of intervention

a)

Study or Subgroup	Intervention Mean	Intervention SD	Intervention Total	Control Mean	Control SD	Control Total	Weight	Mean difference IV, Random, 95% CI
**1.3.1 Community engagement in two research stages**								
Black et al., 2010	0.73	1.16	91	0.61	1.14	93	8.6%	0.12 [-0.21 , 0.45]
Crespo et al., 2012 (Community + Home)	0.96	1.06	196	0.99	1.12	69	9.9%	-0.03 [-0.33 , 0.27]
Crespo et al., 2012 (Community only)	0.85	1.09	145	0.99	1.12	68	9.1%	-0.14 [-0.46 , 0.18]
Crespo et al., 2012 (Home only)	0.95	1.13	167	0.99	1.12	68	9.3%	-0.04 [-0.36 , 0.28]
Gómez et al., 2018	0.64	1.12	974	0.57	1.15	1112	32.3%	0.07 [-0.03 , 0.17]
Peña et al., 2021	0.92	1.15	1544	1.07	1.19	653	30.6%	-0.15 [-0.26 , -0.04]
Wong et al., 2016	1.42	17.819091	288	1.41	14.495282	187	0.1%	0.01 [-2.91 , 2.93]
**Subtotal (95% CI)**			**3405**			**2250**	**100.0%**	**-0.03 [-0.14 , 0.08]**
Heterogeneity: Tau² = 0.01; Chi² = 10.04, df = 6 (P = 0.12); I² = 40%								
Test for overall effect: Z = 0.58 (P = 0.56)								
**1.3.2 Community engagement in three research stages**								
Davis et al., 2016	0.81	1.170299	214	0.8	1.165221	229	4.8%	0.01 [-0.21 , 0.23]
Fotu et al., 2011	1.1	0.9	815	1.1	0.9	897	29.9%	0.00 [-0.09 , 0.09]
Fulkerson et al., 2022	0.69	1	56	1.03	0.89	46	1.7%	-0.34 [-0.71 , 0.03]
Millar et al., 2011	0.5	1.05	1276	0.53	0.95	778	28.1%	-0.03 [-0.12 , 0.06]
Sanigorski et al., 2008	0.54	0.94	833	0.58	0.88	974	30.5%	-0.04 [-0.12 , 0.04]
Scherr et al., 2017	0.53	1.04	229	0.41	1.1	172	5.0%	0.12 [-0.09 , 0.33]
**Subtotal (95% CI)**			**3423**			**3096**	**100.0%**	**-0.02 [-0.07 , 0.03]**
Heterogeneity: Tau² = 0.00; Chi² = 5.14, df = 5 (P = 0.40); I² = 3%								
Test for overall effect: Z = 0.81 (P = 0.42)								
**1.3.3 Community engagement in four research stages**								
White et al., 2019	0.82	1.043552	90	0.65	0.857832	35	100.0%	0.17 [-0.19 , 0.53]
**Subtotal (95% CI)**			**90**			**35**	**100.0%**	**0.17 [-0.19 , 0.53]**
Heterogeneity: Not applicable								
Test for overall effect: Z = 0.93 (P = 0.35)								
Test for subgroup differences: Chi² = 1.14, df = 2 (P = 0.57), I² = 0%								

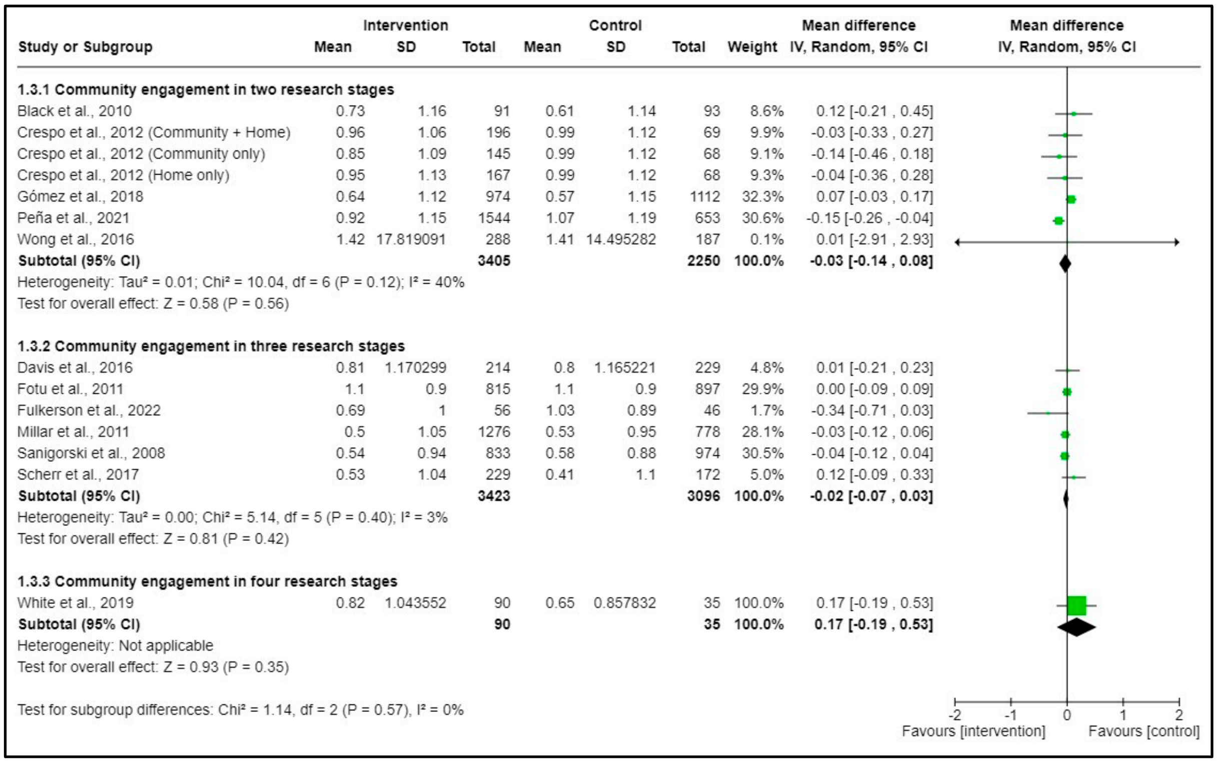

b)

Study or Subgroup	Intervention Mean	Intervention SD	Intervention Total	Control Mean	Control SD	Control Total	Weight	Mean difference IV, Random, 95% CI
**1.5.1 Behavioural change**								
Black et al., 2010	0.73	1.16	91	0.61	1.14	93	7.4%	0.12 [-0.21 , 0.45]
Gómez et al., 2018	0.64	1.12	974	0.57	1.15	1112	86.1%	0.07 [-0.03 , 0.17]
White et al., 2019	0.82	1.043552	90	0.65	0.857832	35	6.4%	0.17 [-0.19 , 0.53]
Wong et al., 2016	1.42	17.819091	288	1.41	14.495282	187	0.1%	0.01 [-2.91 , 2.93]
**Subtotal (95% CI)**			**1443**			**1427**	**100.0%**	**0.08 [-0.01 , 0.17]**
Heterogeneity: Tau² = 0.00; Chi² = 0.34, df = 3 (P = 0.95); I² = 0%								
Test for overall effect: Z = 1.73 (P = 0.08)								
**1.5.2 Environmental change**								
Crespo et al., 2012 (Community + Home)	0.96	1.06	196	0.99	1.12	69	3.7%	-0.03 [-0.33 , 0.27]
Crespo et al., 2012 (Community only)	0.85	1.09	145	0.99	1.12	68	3.3%	-0.14 [-0.46 , 0.18]
Crespo et al., 2012 (Home only)	0.95	1.13	167	0.99	1.12	68	3.4%	-0.04 [-0.36 , 0.28]
Fotu et al., 2011	1.1	0.9	815	1.1	0.9	897	46.3%	0.00 [-0.09 , 0.09]
Millar et al., 2011	0.5	1.05	1276	0.53	0.95	778	43.4%	-0.03 [-0.12 , 0.06]
**Subtotal (95% CI)**			**2599**			**1880**	**100.0%**	**-0.02 [-0.08 , 0.04]**
Heterogeneity: Tau² = 0.00; Chi² = 0.82, df = 4 (P = 0.94); I² = 0%								
Test for overall effect: Z = 0.68 (P = 0.50)								
**1.5.3 Behavioural and Environmental change**								
Davis et al., 2016	0.81	1.170299	214	0.8	1.165221	229	14.8%	0.01 [-0.21 , 0.23]
Fulkerson et al., 2022	0.69	1	56	1.03	0.89	46	6.7%	-0.34 [-0.71 , 0.03]
Peña et al., 2021	0.92	1.15	1544	1.07	1.19	653	29.5%	-0.15 [-0.26 , -0.04]
Sanigorski et al., 2008	0.54	0.94	833	0.58	0.88	974	33.8%	-0.04 [-0.12 , 0.04]
Scherr et al., 2017	0.53	1.04	229	0.41	1.1	172	15.2%	0.12 [-0.09 , 0.33]
**Subtotal (95% CI)**			**2876**			**2074**	**100.0%**	**-0.06 [-0.16 , 0.04]**
Heterogeneity: Tau² = 0.01; Chi² = 8.25, df = 4 (P = 0.08); I² = 52%								
Test for overall effect: Z = 1.16 (P = 0.25)								
Test for subgroup differences: Chi² = 4.78, df = 2 (P = 0.09), I² = 58.1%								

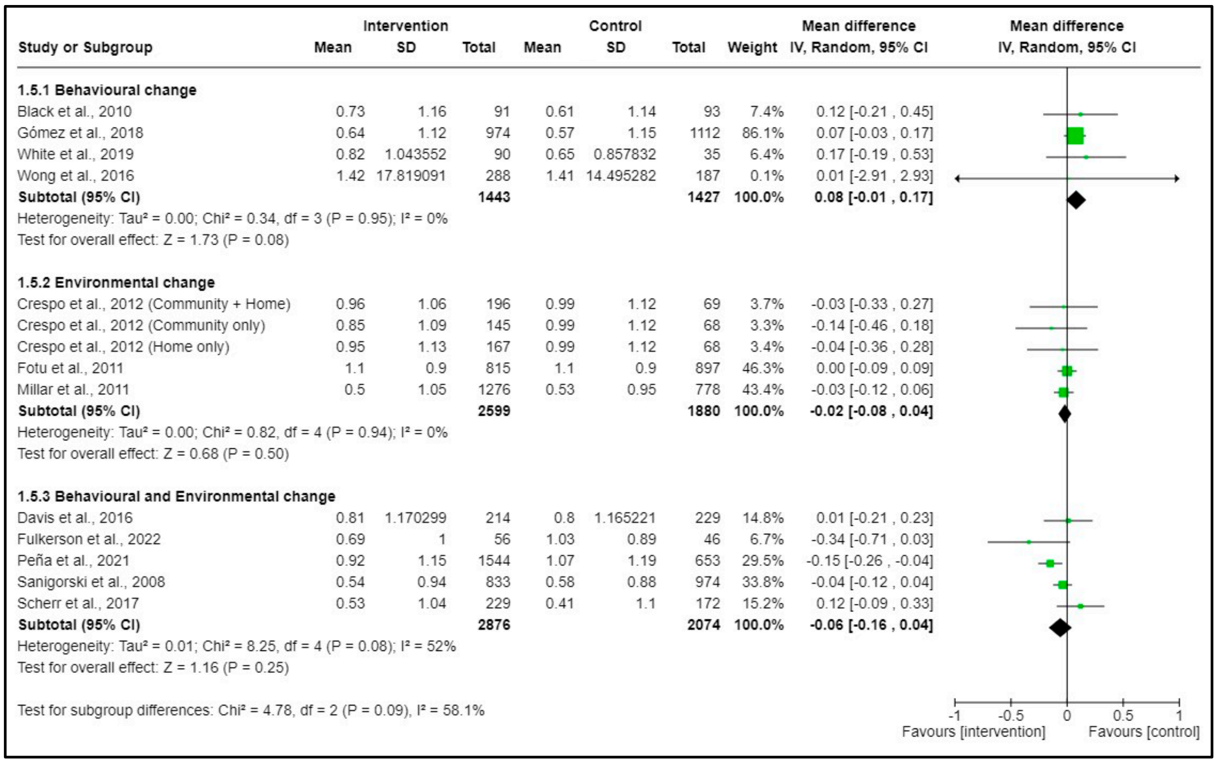

Figure S3. Forest plot of fruit intake (dichotomous data) at post-intervention in three included studies

	Intervention		Control			Risk ratio
**Study or Subgroup**	**Events**	**Total**	**Events**	**Total**	**Weight**	**M-H, Random, 95% CI**
Kremer et al., 2011	241	879	654	2069	26.9%	0.87 [0.77 , 0.98]
Malakellis et al., 2017	326	486	99	148	25.5%	1.00 [0.88 , 1.14]
Millar et al., 2011	685	1276	438	778	47.7%	0.95 [0.88 , 1.03]
**Total (95% CI)**		**2641**		**2995**	**100.0%**	**0.94 [0.87 , 1.02]**
Total events:	1252		1191			

Heterogeneity: Tau² = 0.00; Chi² = 2.89, df = 2 (P = 0.24); I² = 31%
Test for overall effect: Z = 1.57 (P = 0.12)
Test for subgroup differences: Not applicable

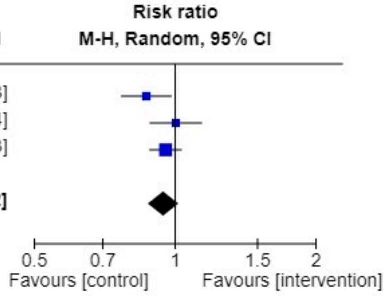

Figure S4. Forest plot of fruit intake (continuous data) at post-intervention in three included studies

Study or Subgroup	Intervention Mean	Intervention SD	Intervention Total	Control Mean	Control SD	Control Total	Weight	Std. mean difference IV, Random, 95% CI
Black et al., 2010	1.2	1.2	91	1.4	1.4	93	38.0%	-0.15 [-0.44 , 0.14]
Fulkerson et al., 2022	0.28	1.41	54	-0.02	0.95	44	22.7%	0.24 [-0.16 , 0.64]
Scherr et al., 2017	1.76	1.43	109	1.87	1.42	86	39.3%	-0.08 [-0.36 , 0.21]
**Total (95% CI)**			**254**			**223**	**100.0%**	**-0.03 [-0.24 , 0.17]**

Heterogeneity: $Tau^2 = 0.01$; $Chi^2 = 2.57$, df = 2 (P = 0.28); $I^2 = 22\%$
Test for overall effect: Z = 0.31 (P = 0.75)
Test for subgroup differences: Not applicable

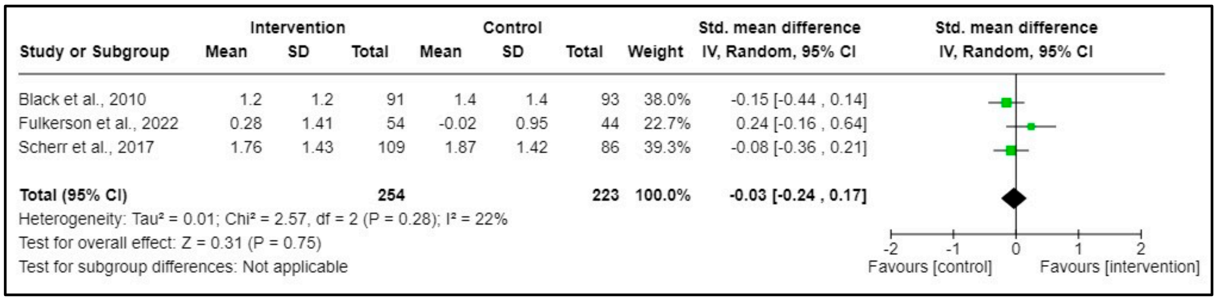

Figure S5. Forest plot of fruit intake (continuous data) at post-intervention according to a) number of community engagement research stages, and b) type of intervention

a)

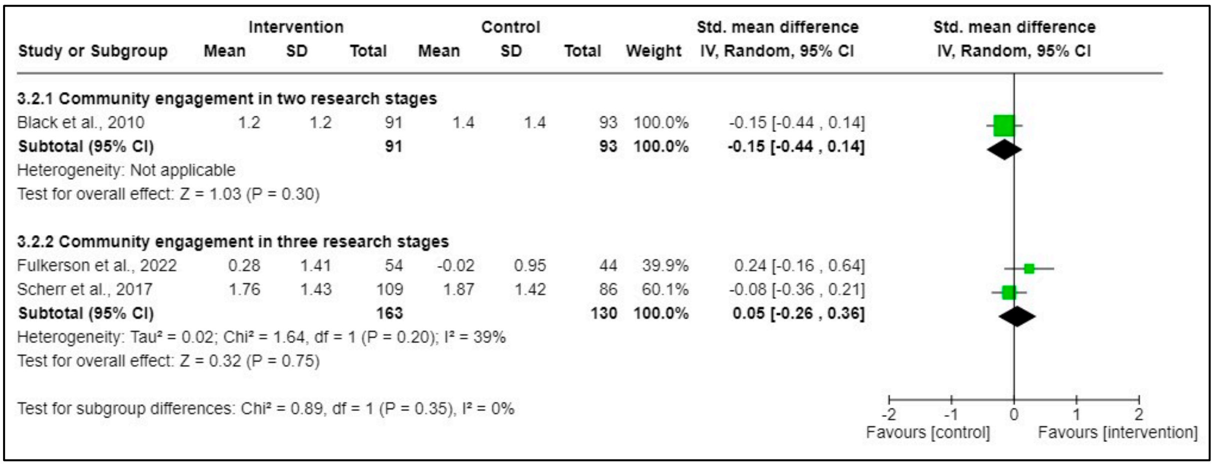

b)

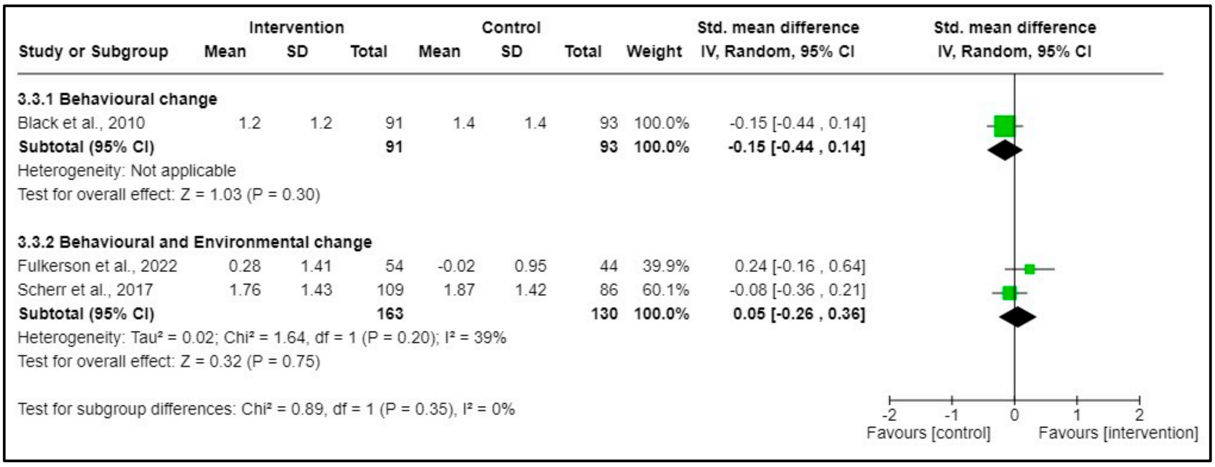

Figure S6. Forest plot of vegetable intake (dichotomous data) at post-intervention in three included studies

Study or Subgroup	Intervention Events	Intervention Total	Control Events	Control Total	Weight	Risk ratio M-H, Random, 95% CI
Kremer et al., 2011	457	879	1179	2069	27.9%	0.91 [0.85 , 0.98]
Malakellis et al., 2017	69	486	16	148	0.6%	1.31 [0.79 , 2.19]
Millar et al., 2011	960	1276	633	778	71.5%	0.92 [0.88 , 0.97]
**Total (95% CI)**		**2641**		**2995**	**100.0%**	**0.92 [0.89 , 0.96]**
Total events:	1486		1828			

Heterogeneity: Tau² = 0.00; Chi² = 1.94, df = 2 (P = 0.38); I² = 0%
Test for overall effect: Z = 4.03 (P < 0.0001)
Test for subgroup differences: Not applicable

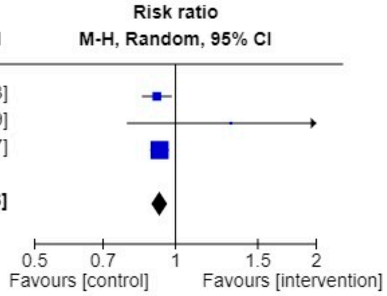

Figure S7. Forest plot of vegetable intake (continuous data) at post-intervention in three included studies

Study or Subgroup	Intervention Mean	Intervention SD	Intervention Total	Control Mean	Control SD	Control Total	Weight	Std. mean difference IV, Random, 95% CI
Black et al., 2010	2	2	91	1.9	1.9	93	37.9%	0.05 [-0.24 , 0.34]
Fulkerson et al., 2022	-0.27	0.94	54	-0.01	0.69	44	23.1%	-0.31 [-0.71 , 0.09]
Scherr et al., 2017	0.81	0.79	109	0.75	0.6	86	39.0%	0.08 [-0.20 , 0.37]
**Total (95% CI)**			**254**			**223**	**100.0%**	**-0.02 [-0.23 , 0.19]**

Heterogeneity: Tau² = 0.01; Chi² = 2.72, df = 2 (P = 0.26); I² = 27%
Test for overall effect: Z = 0.18 (P = 0.86)
Test for subgroup differences: Not applicable

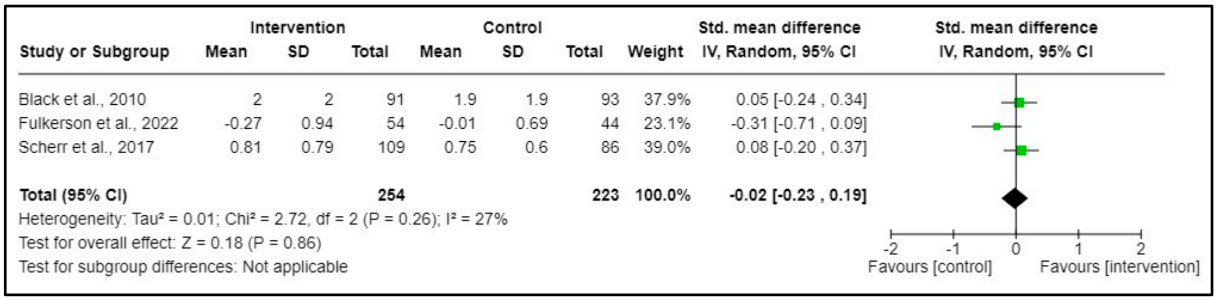

Figure S8. Forest plot of vegetable intake (continuous data) at post-intervention according to a) number of community engagement research stages, and b) type of intervention

a)

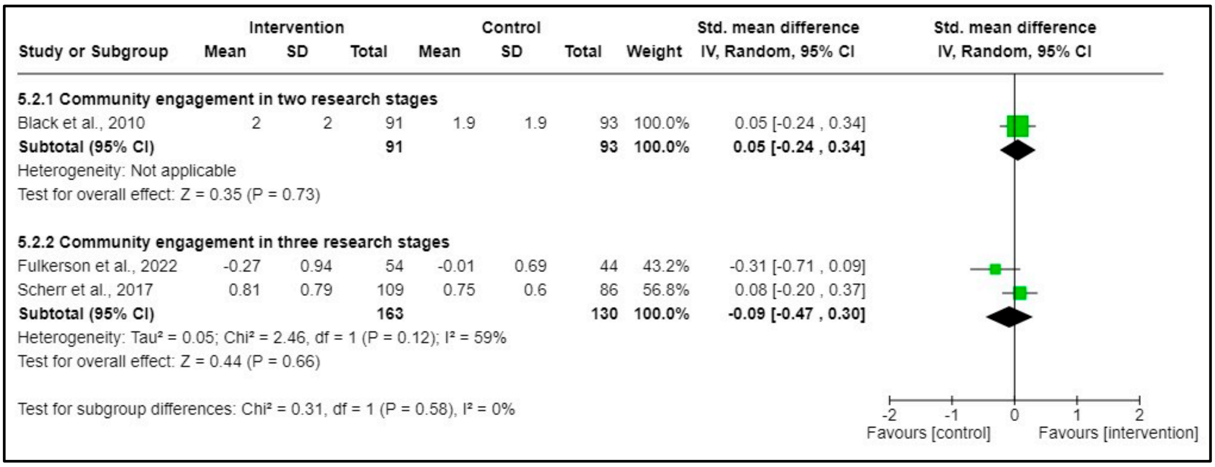

b)

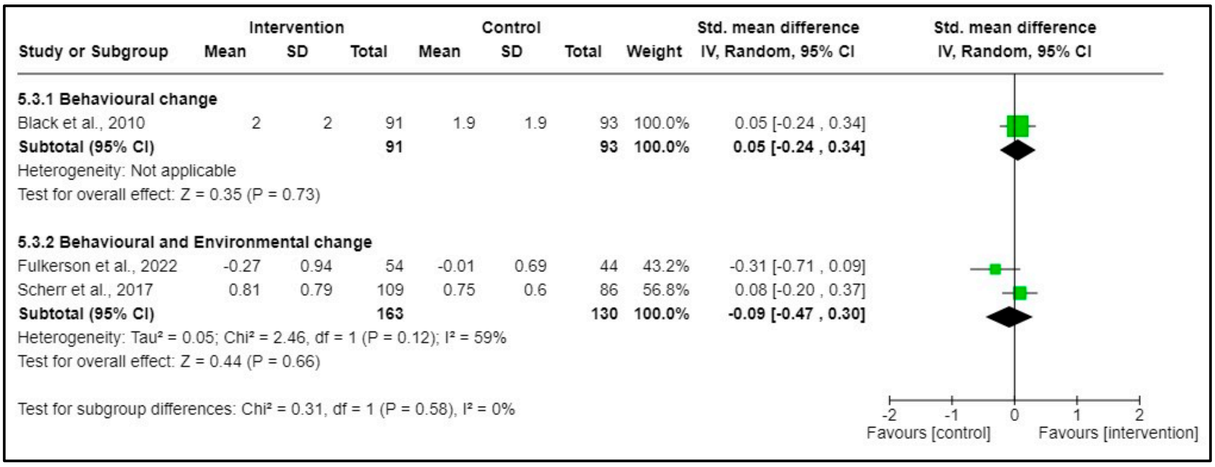

Figure S9. Forest plot of sugar-sweetened beverage intake (dichotomous data) at post-intervention in four included studies

	Intervention		Control			Risk ratio
**Study or Subgroup**	**Events**	**Total**	**Events**	**Total**	**Weight**	**M-H, Random, 95% CI**
Fotu et al., 2011	74	227	187	437	16.4%	0.76 [0.61 , 0.95]
Kremer et al., 2011	735	879	1730	2069	31.4%	1.00 [0.97 , 1.04]
Malakellis et al., 2017	325	486	94	148	23.2%	1.05 [0.92 , 1.21]
Millar et al., 2011	709	1276	504	778	29.1%	0.86 [0.80 , 0.92]
**Total (95% CI)**		**2868**		**3432**	**100.0%**	**0.93 [0.82 , 1.05]**
Total events:	1843		2515			

Heterogeneity: Tau² = 0.01; Chi² = 24.11, df = 3 (P < 0.0001); I² = 88%

Test for overall effect: Z = 1.21 (P = 0.22)

Test for subgroup differences: Not applicable

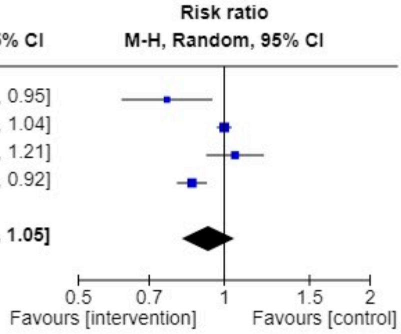

Figure S10. Forest plot of sugar-sweetened beverage intake (continuous data) at post-intervention in three included studies

Study or Subgroup	Intervention Mean	Intervention SD	Intervention Total	Control Mean	Control SD	Control Total	Weight	Std. mean difference IV, Random, 95% CI
Black et al., 2010	0.6	0.7	91	0.7	0.7	93	18.6%	-0.14 [-0.43 , 0.15]
Crespo et al., 2012 (Community + Home)	0.39	1.12	145	0.88	1.65	69	18.7%	-0.37 [-0.66 , -0.08]
Crespo et al., 2012 (Community only)	0.56	1.26	196	0.88	1.65	68	20.4%	-0.23 [-0.51 , 0.04]
Crespo et al., 2012 (Home only)	0.58	1.3	167	0.88	1.65	68	19.5%	-0.21 [-0.49 , 0.07]
Folta et al., 2013 (Secondary Outcomes)	5.5	6.7	72	7.6	7	265	22.8%	-0.30 [-0.56 , -0.04]
**Total (95% CI)**			**671**			**563**	**100.0%**	**-0.25 [-0.38 , -0.13]**

Heterogeneity: Tau² = 0.00; Chi² = 1.45, df = 4 (P = 0.84); I² = 0%

Test for overall effect: Z = 3.98 (P < 0.0001)

Test for subgroup differences: Not applicable

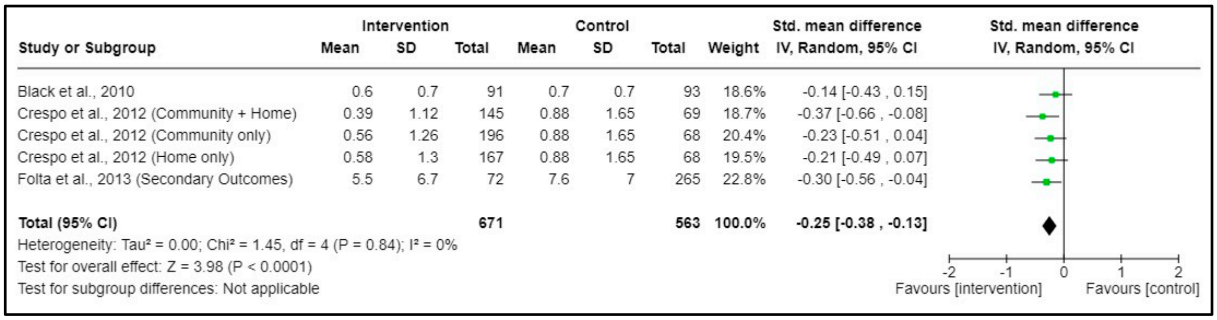

Figure S11. Forest plot of sugar-sweetened beverage intake (continuous data) at post-intervention according to a) number of community engagement research stages, and b) type of intervention

a)

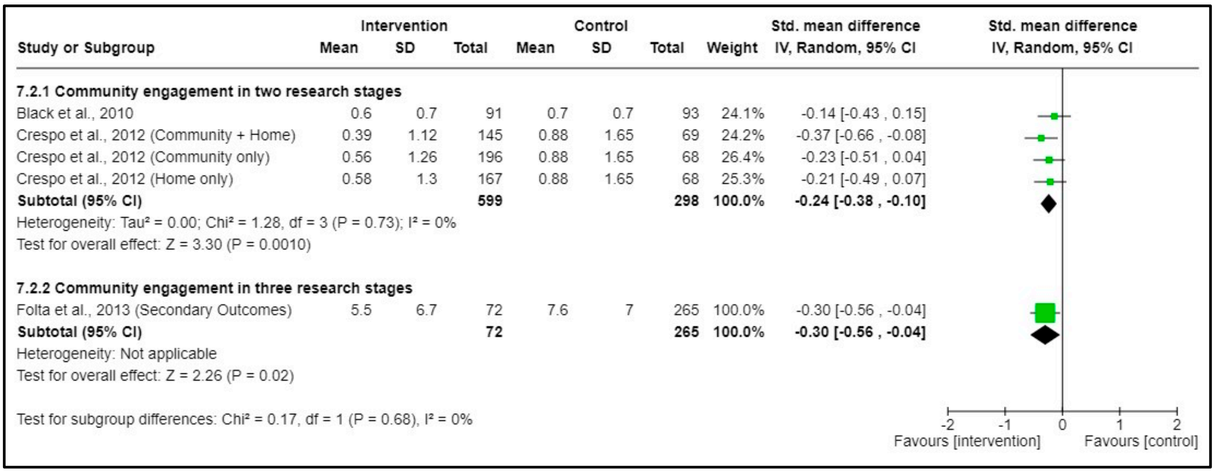

b)

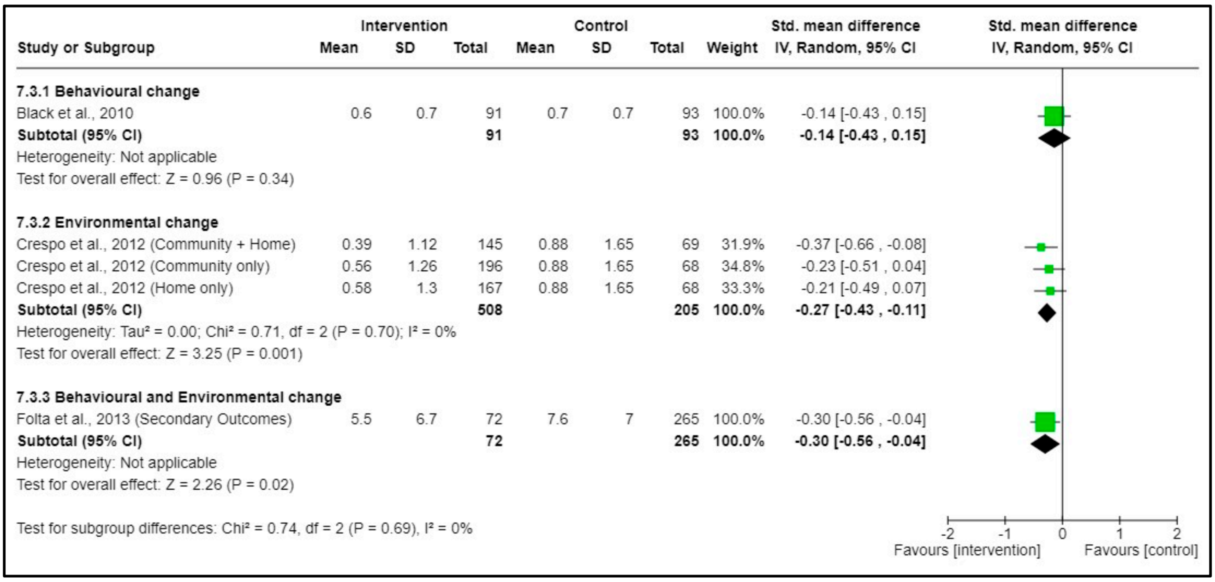

Figure S12. Forest plot of MVPA (continuous data) at post-intervention in four included studies

Study or Subgroup	Intervention Mean	Intervention SD	Intervention Total	Control Mean	Control SD	Control Total	Weight	Std. mean difference IV, Random, 95% CI
Adab et al., 2018	68.2492	47.7448	492	66.1986	41.6716	560	64.9%	0.05 [-0.08 , 0.17]
Fulkerson et al., 2022	-0.1514285714	3.0671428571	51	0.1728571429	3.2871428571	40	5.5%	-0.10 [-0.52 , 0.31]
Kattelmann et al., 2019 (Secondary Outcomes)	52.8	29.0712	25	64.8	24.401304	8	1.5%	-0.42 [-1.22 , 0.39]
Wong et al., 2016 (MPA)	3.9	13.2	144	5.4	1.1	93	14.0%	-0.14 [-0.41 , 0.12]
Wong et al., 2016 (VPA)	0.07	16.68	144	0.09	3.878144	94	14.1%	-0.00 [-0.26 , 0.26]
**Total (95% CI)**			**856**			**795**	**100.0%**	**-0.00 [-0.10 , 0.10]**

Heterogeneity: Tau² = 0.00; Chi² = 3.00, df = 4 (P = 0.56); I² = 0%
Test for overall effect: Z = 0.05 (P = 0.96)
Test for subgroup differences: Not applicable

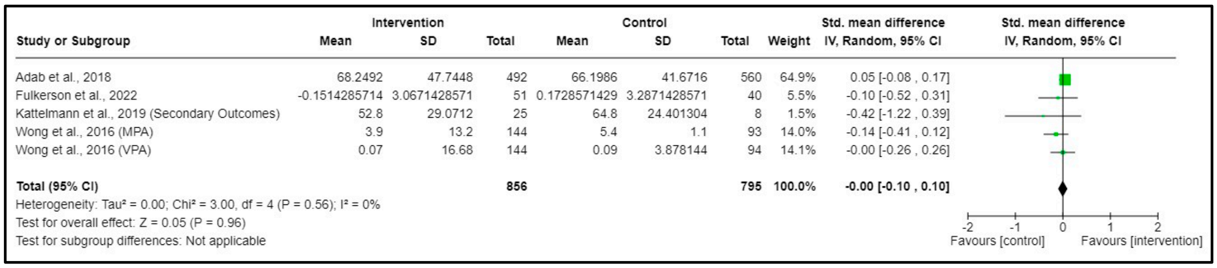

Figure S13. Forest plot of MVPA (continuous data) at post-intervention according to a) number of community engagement research stages, and b) type of intervention

a)

Study or Subgroup	Intervention Mean	Intervention SD	Intervention Total	Control Mean	Control SD	Control Total	Weight	Std. mean difference IV, Random, 95% CI
**8.2.1 Community engagement in two research stages**								
Wong et al., 2016 (MPA)	3.9	13.2	144	5.4	1.1	93	49.8%	-0.14 [-0.41 , 0.12]
Wong et al., 2016 (VPA)	0.07	16.68	144	0.09	3.878144	94	50.2%	-0.00 [-0.26 , 0.26]
**Subtotal (95% CI)**			**288**			**187**	**100.0%**	**-0.07 [-0.26 , 0.11]**
Heterogeneity: Tau² = 0.00; Chi² = 0.58, df = 1 (P = 0.45); I² = 0%								
Test for overall effect: Z = 0.78 (P = 0.44)								
**8.2.2 Community engagement in three research stages**								
Adab et al., 2018	68.2492	47.7448	1134	66.1986	41.6716	1328	96.5%	0.05 [-0.03 , 0.13]
Fulkerson et al., 2022	-0.1514285714	3.0671428571	51	0.1728571429	3.2871428571	40	3.5%	-0.10 [-0.52 , 0.31]
**Subtotal (95% CI)**			**1185**			**1368**	**100.0%**	**0.04 [-0.04 , 0.12]**
Heterogeneity: Tau² = 0.00; Chi² = 0.47, df = 1 (P = 0.49); I² = 0%								
Test for overall effect: Z = 1.03 (P = 0.30)								
**8.2.3 Community engagement in four research stages**								
Kattelmann et al., 2019 (Secondary Outcomes)	52.8	29.0712	25	64.8	24.401304	8	100.0%	-0.42 [-1.22 , 0.39]
**Subtotal (95% CI)**			**25**			**8**	**100.0%**	**-0.42 [-1.22 , 0.39]**
Heterogeneity: Not applicable								
Test for overall effect: Z = 1.02 (P = 0.31)								
Test for subgroup differences: Chi² = 2.39, df = 2 (P = 0.30), I² = 16.2%								

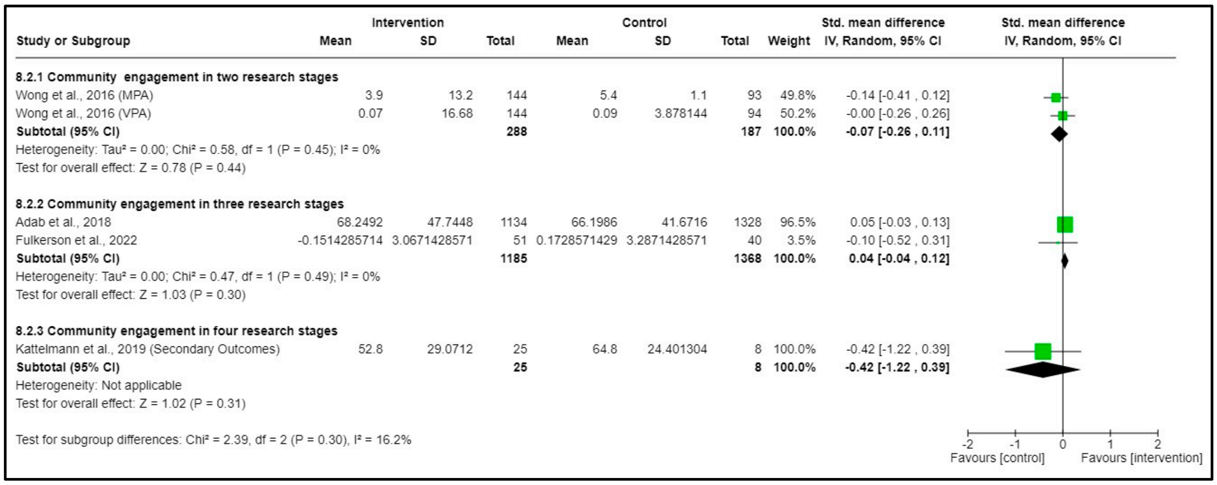

b)

Study or Subgroup	Intervention Mean	Intervention SD	Intervention Total	Control Mean	Control SD	Control Total	Weight	Std. mean difference IV, Random, 95% CI
**8.3.1 Behavioural change**								
Adab et al., 2018	68.2492	47.7448	1134	66.1986	41.6716	1328	80.4%	0.05 [-0.03 , 0.13]
Kattelmann et al., 2019 (Secondary Outcomes)	52.8	29.0712	25	64.8	24.401304	8	1.0%	-0.42 [-1.22 , 0.39]
Wong et al., 2016 (MPA)	3.9	13.2	144	5.4	1.1	93	9.3%	-0.14 [-0.41 , 0.12]
Wong et al., 2016 (VPA)	0.07	16.68	144	0.09	3.878144	94	9.3%	-0.00 [-0.26 , 0.26]
**Subtotal (95% CI)**			**1447**			**1523**	**100.0%**	**0.02 [-0.06 , 0.10]**
Heterogeneity: Tau² = 0.00; Chi² = 3.10, df = 3 (P = 0.38); I² = 3%								
Test for overall effect: Z = 0.47 (P = 0.64)								
**8.3.2 Behavioural and Environmental change**								
Fulkerson et al., 2022	-0.1514285714	3.0671428571	51	0.1728571429	3.2871428571	40	100.0%	-0.10 [-0.52 , 0.31]
**Subtotal (95% CI)**			**51**			**40**	**100.0%**	**-0.10 [-0.52 , 0.31]**
Heterogeneity: Not applicable								
Test for overall effect: Z = 0.48 (P = 0.63)								
Test for subgroup differences: Chi² = 0.31, df = 1 (P = 0.57), I² = 0%								

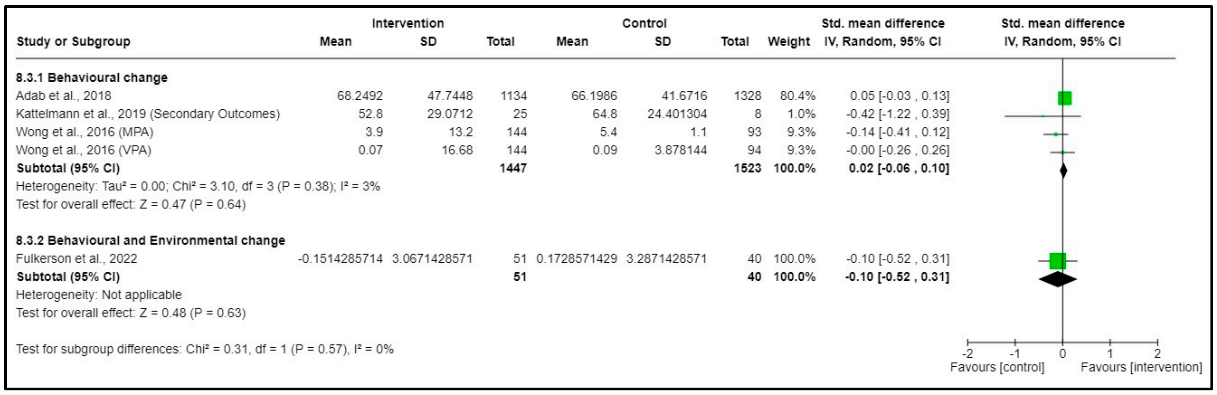

Figure S14. Forest plot of small screen time (dichotomous data) at post-intervention in four included studies

Study or Subgroup	Intervention Events	Intervention Total	Control Events	Control Total	Weight	Risk ratio M-H, Random, 95% CI
Fotu et al., 2011	135	163	351	437	25.5%	1.03 [0.95 , 1.12]
Kremer et al., 2011	532	879	1473	2069	28.0%	0.85 [0.80 , 0.90]
Malakellis et al., 2017	306	486	83	148	17.3%	1.12 [0.96 , 1.31]
Millar et al., 2011	938	1276	640	778	29.2%	0.89 [0.85 , 0.94]
**Total (95% CI)**		**2804**		**3432**	**100.0%**	**0.95 [0.86 , 1.05]**
Total events:	1911		2547			

Heterogeneity: Tau² = 0.01; Chi² = 21.02, df = 3 (P = 0.0001); I² = 86%

Test for overall effect: Z = 1.01 (P = 0.31)

Test for subgroup differences: Not applicable

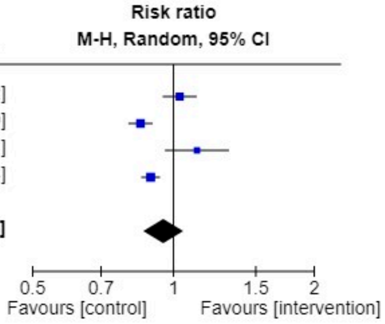

Figure S15. Forest plot of electronic game time (dichotomous data) at post-intervention in four included studies

Study or Subgroup	Intervention Events	Intervention Total	Control Events	Control Total	Weight	Risk ratio M-H, Random, 95% CI
Fotu et al., 2011	133	163	331	437	26.4%	1.08 [0.98 , 1.18]
Kremer et al., 2011	488	879	1248	2069	27.6%	0.92 [0.86 , 0.99]
Malakellis et al., 2017	215	486	67	148	18.4%	0.98 [0.80 , 1.20]
Millar et al., 2011	699	1276	537	778	27.6%	0.79 [0.74 , 0.85]
**Total (95% CI)**		**2804**		**3432**	**100.0%**	**0.93 [0.81 , 1.07]**
Total events:	1535		2183			

Heterogeneity: Tau² = 0.02; Chi² = 29.64, df = 3 (P < 0.00001); I² = 90%

Test for overall effect: Z = 0.98 (P = 0.33)

Test for subgroup differences: Not applicable

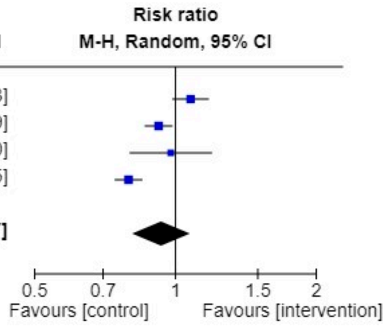

Figure S16. Funnel plot of BMI-z using standard error (SE) against mean difference (MD): 12 studies (lower MD value indicates better outcome)

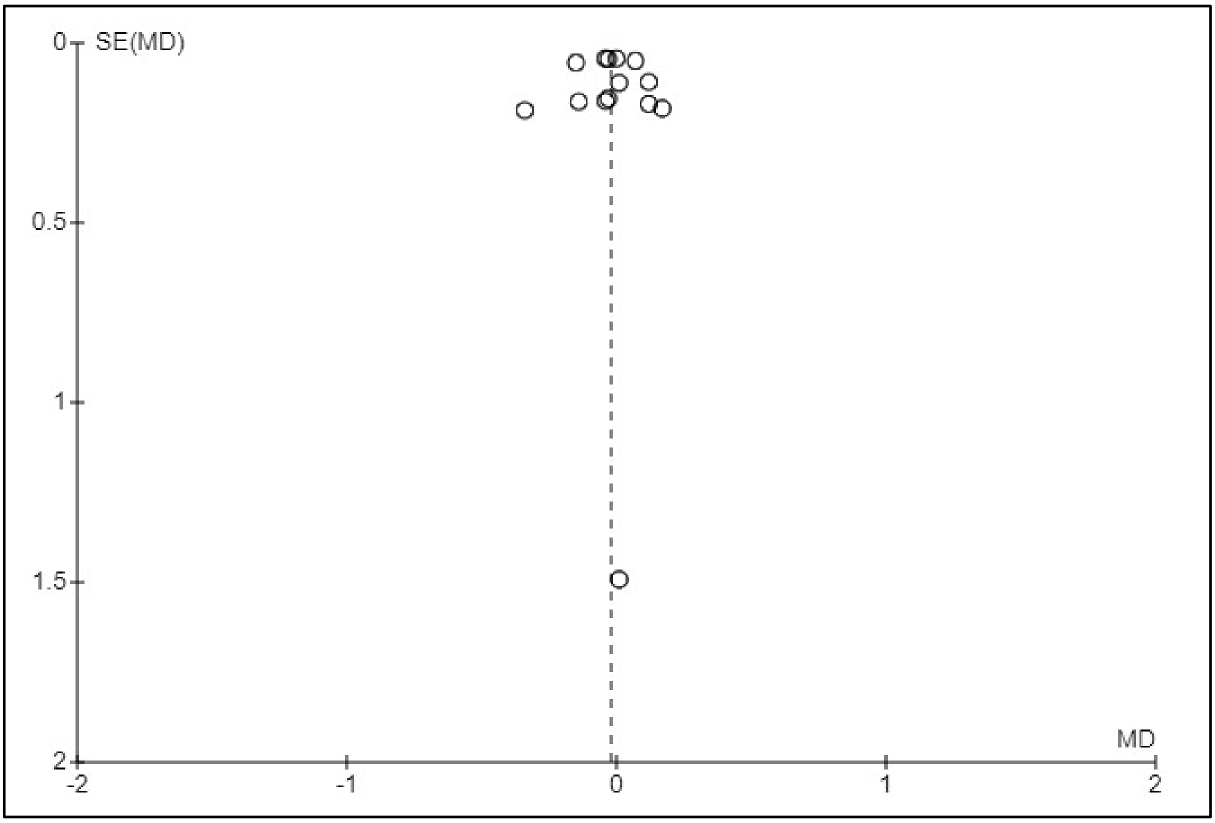

Supplement: Supplementary file 1 [file nutrients-16-03465-s001.zip › Supplementary File S2.pdf]
